# Evaluation of a Positive Psychological Intervention to Reduce Work Stress among Rural Community Health Workers in India: Results from a Randomized Pilot Study

**DOI:** 10.1007/s10902-024-00852-6

**Published:** 2025-02-17

**Authors:** Ameya P. Bondre, Spriha Singh, Abhishek Singh, Aashish Ranjan, Azaz Khan, Lochan Sharma, Dinesh Bari, G Sai Teja, Laxmi Verma, Mehak Jolly, Payal Pandit, Radhika Sharma, Ritu Dangi, Romi Ahuja, Sneha Rani Nayak, Surbhi Agrawal, Jyotsna Agrawal, Seema Mehrotra, Rahul Shidhaye, Anant Bhan, John A. Naslund, Steve D. Hollon, Deepak Tugnawat

**Affiliations:** 1 Sangath Bhopal Hub, 106, Good Shepherd Colony, Kolar Road, Bhopal, Madhya Pradesh 462042 India; 2 National Institute of Mental Health and Neurosciences, Hosur Road, Lakkasandra, Wilson Garden, Bengaluru, Karnataka 560029 India; 3 Pravara Institute of Medical Sciences, Rahata, Ahmednagar, Maharashtra 413736 India; 4 Department of Global Health and Social Medicine, Harvard Medical School, 25 Shattuck St, Boston, MA 02115 United States; 5 Department of Psychology, Vanderbilt University, Nashville, TN 37023 United States

**Keywords:** Character strengths, Work stress, Burnout, Happiness, Community health workers, India

## Abstract

Efforts to reduce work stress among frontline health workers in India, namely the rural Accredited Social Health Activists or ‘ASHAs’ (resident women, lay health workers) have predominantly emphasized on ‘extrinsic’ variables (e.g., financial incentives, or enhanced supervision), with little consideration of ‘intrinsic’ factors like the wellbeing of ASHAs themselves or building their abilities in managing work stress. ‘Character-strengths’ based positive psychological interventions have shown improvements in work wellbeing and engagement, but these findings have been largely observed in the Global North, with no experimental studies in India. This study is a pilot randomized controlled trial where rural ASHAs were allocated to receive regular supervision by their supervisors appointed by the health system (control arm), or a character-strengths based coaching intervention in addition to regular supervision (intervention arm). The intervention included a residential workshop and follow-on weekly individual telephonic coaching support. We aimed to examine the preliminary effectiveness of the intervention on happiness of ASHAs using the Authentic Happiness Inventory (primary outcome). At 3-month follow-up, ASHAs in the intervention arm showed higher total happiness scores (n = 30, Mean = 83.6; SD = 13.32) than ASHAs in the control arm (n = 31, Mean = 76.32; SD = 13.16), with a significant between-arm difference (Cohen’s d: 0.55). Secondary outcomes (e.g., burnout, motivation) showed non-significant between-arm differences at follow-up. We conducted telephone-interviews of ASHAs to gather their experiences of the intervention and used thematic analysis to assess its feasibility and acceptability. This study critically contributes to inform the ways by which rural frontline workers can use character-strengths to improve work wellbeing in low-resource settings.

**Supplementary Information:**

The online version contains supplementary material available at 10.1007/s10902-024-00852-6.

## Introduction

In low-resource settings worldwide, community health workers (CHWs) often experience stress due to substantial workloads and inadequate compensation, within the overarching backdrop of poverty and their lower hierarchical positions within health systems, and gender inequality for female CHWs (Dugani et al., 2018; Li et al., 2014; Selamu et al., 2017, 2019). In India, these factors apply to the more than one million female lay health care providers called ‘Accredited Social Health Activists (ASHAs),’ who deliver a range of preventive and curative primary care services in urban and rural settings (Scott et al., 2019). At the village-level, ASHAs, who are predominantly married women residing in the communities they serve, function as ‘voluntary’ workers (i.e., unsalaried, receiving performance incentives) and intermediaries between the local communities and formal primary healthcare clinics (Ministry of Health and Family Welfare, 2005). However, they shoulder a range of responsibilities such as building awareness on infectious disease control and prevention, and sanitation; antenatal, intra-natal and postnatal services; counselling on family planning and safe abortion; immunization and vitamin A supplementation for children; promotion of early and exclusive breastfeeding, birth spacing, and postnatal care; in addition to conducting household surveys, and screening of non-communicable diseases (Shrivastava et al., 2023).

With these responsibilities, the ASHA cadre is experiencing high levels of stress and burnout (Gopalan et al., 2012; Muke et al., 2019; Pulagam & Satyanarayana, 2021; Tripathy et al., 2016). The reasons are manifold: their involvement in multiple programs, lowest hierarchical positioning, low and untimely monetary incentives, role confusion, unmet capacity-building needs, limited educational qualifications, working within inadequate infrastructures and centralized governance mechanisms, and the larger social realities of poverty, and patriarchy (Kawade et al., 2021; Manjunath et al., 2022; Muke et al., 2019; Oswal et al., 2020; Pulagam & Satyanarayana, 2021; Saprii et al., 2015; Scott & Shanker, 2010; Scott et al., 2019; Sharma et al., 2014; Shet et al., 2018; Ved et al., 2019). Efforts to address these issues have predominantly emphasized on ‘extrinsic’ variables, for example, financial incentives (Bhattacharyya et al., 2001), enhanced supervisory approaches (Bajpai et al., 2011; Mondal & Murhekar, 2018) or flexible task assignments (Bhatia, 2014). While these efforts are important, there has been inadequate consideration of ‘intrinsic’ factors like the happiness and work well being of ASHAs themselves, or their abilities in managing stress and burnout (Pandey & Singh, 2015, 2016; Wahid et al., 2020). ASHAs incur ‘emotional labor’ (Pandey & Singh, 2015) as they adjust their emotions in response to evolving work-related and situational demands, which may contribute to their perceptions of inadequacy or lack of support and reduce their emotional wellbeing.

Positive Psychological Interventions (PPIs) have shown to cultivate positive emotions, behaviors, and/or thoughts by identifying and leveraging an individual’s positive traits to effectively manage stress (Seligman & Csikszentmihalyi, 2000). PPIs have been applied to workplace settings to encompass deliberate activities incorporating three foundational pillars: positive subjective experience, positive character traits, and positive institutions (Meyers et al., 2013). More recently, for nurses (Luo et al., 2019), whose work closely relates to ASHAs (Pandey & Singh, 2015), and other healthcare professionals (nurses and social workers, amongst others) with depression, anxiety and work stress, PPIs have shown positive work-related outcomes such as reduction in burnout, and decrease in depression, anxiety, stress, and emotional exhaustion scores, as well as increase in satisfaction with life, respectively (Donaldson et al., 2019). While positive psychology interventions cannot possibly address the broader structural/systemic challenges impacting ASHAs, they are important to enable ASHAs to manage their stress in ways described above (especially as these interventions have not been piloted in the rural Indian context), as demonstrated for other cadres, and to develop positive mechanisms to cope with work stress.

Briefly, five types of workplace PPIs (Donaldson et al., 2019) have been described: a) psychological capital interventions specifically aim to improve hope, resilience, self-efficacy and optimism; b) job crafting interventions promote proactive behaviors that engage employees to redesign their work, c) character strengths-based interventions promote the ways of behaving, thinking or feeling at an individual level to enhance the natural capacity and/or enjoyment towards work, which allow the individual to achieve optimal functioning while they pursue valued outcomes; d) gratitude interventions help employees appreciate the positive aspects of life and e) employee wellbeing interventions focus on positive work relationships and self-compassion. We conducted a desk review of these PPIs, and considered ASHAs’ low hierarchical position, low pay, and tremendous workloads. We aimed to focus on employee character-strengths (CS) based interventions because compared to other workplace PPIs, CS interventions have shown positive work outcomes over a range of domains like change in burnout, work wellbeing and engagement at work (Donaldson et al., 2019). Further, interventions leveraging personal strengths or having components of CS use, have achieved success in generating positive work outcomes for various target groups such as workplace employees (Harzer & Ruch, 2012; Mackie, 2014; Meyers & Van Woerkom, 2017), nurses (Monzani et al., 2021) and community health workers (Mathias et al., 2018; Sundar et al., 2016).

However, these studies have largely been conducted in the high-resource or Global North settings. In Indian low-resource settings, wellbeing-enhancing interventions have been scarcely studied within experimental designs or through longitudinal studies (Ghosh & Deb, 2016). Moreover, PPI research so far has been limited to groups other than work-stressed rural health workers in India, like students, adolescents, youth, and general employees (Ghosh & Deb, 2016; Mehrotra, 2013; Nath & Pradhan, 2011; Sundar, 2016; Mathias et al., 2018; Gupta et al., 2014; Murthy, 2014), and without systematically focusing on the use of character-strengths. This gap in literature helped frame the research question for the proposed study—can a structured positive psychological intervention leveraging the use of character strengths reduce work stress and improve wellbeing in rural ASHAs, which constitute a large public health workforce in India?

To address this question, we first developed a character-strengths based coaching intervention to reduce work stress and improve well being of rural ASHAs. The development of our intervention occurred over 11 months, involving (1) formative work (Shrivastava et al., 2023), (2) blueprint development, (3) content development and (4) content-testing (Khan et al., 2023). The intervention involves two sequential components: a 5-day residential workshop on the use of strengths to address work stress and follow-on 8- to 10-weeks of remote telephonic coaching (weekly) to provide consistent individualized support to ASHAs, as they resume work and face stressful situations. Formative focus group discussions and thematic qualitative analysis of ASHA perspectives/feedback were conducted to inform further modifications to the workshop component of the intervention (Khan et al., 2023). During the development process, consistent efforts were made to tailor and contextually adapt the evidence-based character-strengths intervention approaches with iterative feedback from ASHAs. This resulted in the finalization of the coaching material consisting of a ‘content manual’ (for ASHAs) with four modules (Khan et al., 2023), including character-strengths based ‘strategies’ to address challenges/stressors; along with a ‘facilitator manual’ for the coaches.

This study aims to test the hypothesis that a pilot of the above character-strengths based coaching intervention will show preliminary effectiveness to increase the subjective wellbeing (authentic happiness) of ASHAs in rural Madhya Pradesh, India, in comparison to the regular (face-to-face) supportive supervision that ASHAs already receive by their health system-appointed supervisors (control arm). The study also aims to examine the feasibility and acceptability of the strengths-based intervention among the rural ASHAs, with an emphasis on the remote telephonic support component. This study forms the pilot phase of an ongoing larger individual parallel randomized controlled trial—clinical trials.gov identifier: NCT06013488 (ClinicalTrials.gov, 2024) that aims to demonstrate the effectiveness of this positive psychological intervention to improve the wellbeing of rural ASHAs in Madhya Pradesh, India.

## Methods

We report how we determined our sample size (Sect. 2.3), all data exclusions (Sect. 2.4), information on manipulation check (Sect. 2.9.3), and all measures (Sect. 2.8) in the study (Simmons et al., 2012). We have uploaded the de-identified data, code plan and analysis scripts in an online repository. Institutional (ethics) review board at Sangath, India approved all study procedures (details in Sect. 5).

### Study Design

An exploratory two-arm randomized pilot study was conducted, which followed the extension of CONSORT guidelines to pilot studies (Eldridge et al., 2016). The study was conducted from November 2022 to June 2023. ASHAs in both arms received routine supervision provided by the ASHA ‘facilitators’ (government appointed supervisors) and ASHAs in the intervention arm, additionally, received the aforementioned intervention (character-strengths based coaching).

### Setting

ASHAs were recruited from all seven rural blocks of Raisen district (i.e., Badi Barelli, Udaipura, Sanchi, Obedullaganj, Gairatganj, Silwani, and Begumganj) of Madhya Pradesh in central India, to represent the entire district in the simple random sampling of ASHAs (see 2.5). This district was selected because Sangath, the research organization leading this pilot study, has an established Memorandum of Understanding with the Madhya Pradesh state government. Madhya Pradesh is a large, centrally located state with over 72 million people (73% rural) (Office of the Registrar General and Census Commissioner, 2011), and ranks lower than many other Indian states in human development and availability of resources (Menon, 2008; Suryanarayana et al., 2012). Raisen district covers an area of 8,466 sq. km, with a population of ~ 1.3 million, which is predominantly rural (77.2%), and has a male–female sex ratio of 1000:901(Government of Madhya Pradesh, n.d.).

### Participants

**Fig. 1:**
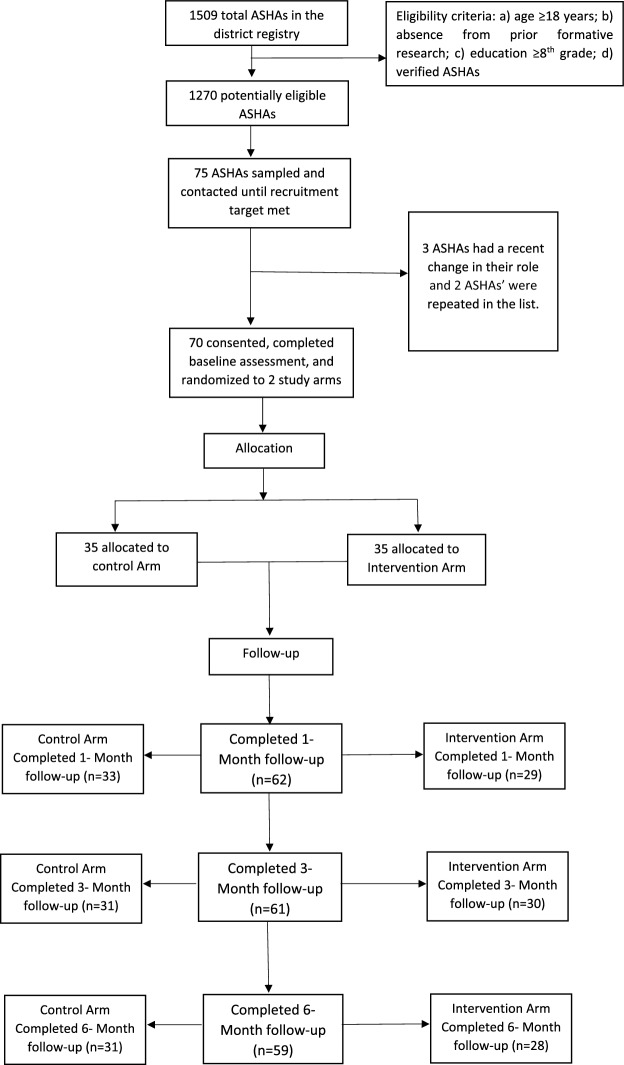
Study consort diagram

We did not have context-specific literature on preliminary effectiveness of the character-strengths based coaching intervention on subjective wellbeing of ASHAs and further, our available time and resources allowed for a sample size of around 70 ASHAs, or roughly three intervention workshop batches of 11–12 ASHAs each. To define the reasonable minimum detectable effect, we conducted an a priori sample size calculation (Lakens, 2022; Zhong, 2009) to hypothesize a 9 point between-arm difference in the mean of the total Authentic Happiness Inventory (AHI) scores at 3-month follow-up (mean AHI score of 75 and 84 for the control and intervention arm respectively, with standard deviation of 13 in each arm). Our higher hypothesized effect size, or a Cohen’s d of 0.69, considered the prevailing work stress in ASHAs and absence of any such stress-reduction interventions provided to them in the past by the health system/other agencies. To achieve this difference at 80% power and 0.05 significance level (two-tailed), we computed a sample size of 34 ASHAs per arm. In practice, we targeted a total sample size of 70 for recruitment and arrived at 61 for analysis (primary outcome at 3-month follow-up) after attrition (13%, see Fig. 1). The targeted sample size was also considered sufficient for achieving our objective of assessing the acceptability and feasibility of the study intervention (Billingham et al., 2013), and we hoped to achieve a significant between-arm difference at 3-month follow-up in the authentic happiness (wellbeing) scores, our measure of preliminary effectiveness.

Each ASHA typically covers a population of ~ 1000 and receives performance- and service-based incentives for facilitating immunization, referral, and escort services for institutional deliveries (Saprii et al., 2015). ASHA facilitators, who were not included as study participants (explained below), provide mentoring and support to ASHAs, and monitor their performance, with one ASHA facilitator typically supervising a group of 10 to 20 ASHAs (National Health Mission, 2005–2012) in face-to-face weekly meetings (both arms).

### Inclusion and Exclusion Criteria

Rural ASHAs working in Raisen district were included in the study if they were -

1.  ≥ 18 Years (the minimum age required for employment by the National Health Mission) (Scott et al., 2019; Ved et al., 2019),
2. Verified i.e., their record was available in the district health staff registry, and they were actively working as confirmed over a check-in phone call made by the research team,
3. Having a minimum education level of up to 8th standard (i.e., in order to read and write, to access the intervention coaching manual and complete the study assessments),
4. Willing to provide written informed consent, and
5. Willing to participate in the study (including the 5-day intervention workshop and 8- to 10-weeks of remote coaching support) and stay in the study area during the study period.

Rural ASHAs were excluded if—

1. They had participated in prior formative research activities (due to prior exposure to content related to the intervention); we confirmed the same through a list of district ASHAs provided by the National Health Mission,
2. They had planned to migrate, or not continue working, or had resigned/planning to change jobs within six months of recruitment,
3. They reported as being unable to use a smartphone (basic smartphone usage ability was required during the remote coaching phase of the intervention), or
4. They had significant speech, sight, or hearing impairment.

ASHA facilitators/supervisors were not included owing to hierarchical differences between them and the ASHAs, with potential effects on absorption of intervention content, and consequently, on their wellbeing (primary outcome of interest).

### Recruitment Procedure

The research team obtained a list of actively working ASHAs in Raisen district from National Health Mission officials (sampling frame). We screened the list containing 1509 verified ASHAs based on certain eligibility criteria such as age (≥ 18 years), education (≥ 8th standard), and absence from earlier formative research activities. We found a total of 1270 potentially eligible ASHAs. From this list, the study data manager randomly selected 75 potentially eligible ASHAs (simple random sampling without replacement) using the Stata software (StataCorp, 2021). Please refer Fig. 1 for the flow of sampling. Research assistants then contacted these ASHAs by phone to confirm their complete eligibility as per the criteria stated in 2.4, willingness, and availability to participate in the study.

The fully eligible ASHAs were invited to attend an information session (in groups on different days) at a nearby primary or community health center to learn more about the study. In this session, the research team described the purpose of the study and informed the ASHAs that the study involved a collaboration with the National Health Mission. The research team also explained what the intervention involved, in addition to the study procedures, and outcome assessments. The research team clarified any queries posed by the ASHAs, and importantly, they explained that their decision to participate was completely voluntary. ASHAs who expressed an interest in participating re-confirmed their eligibility criteria and were provided with an information sheet and individual written-informed consent form. ASHAs were informed that their decision to participate or withdraw from the study at any time would not have any consequences on their current employment as a community health worker, and any data collected during the study would be kept confidential, and no identifiable results would be shared with the health system or others outside the study team. ASHAs who consented, then participated in a subsequent baseline assessment (2.8). Seventy ASHAs were consented and recruited.

### Randomization

Participants were randomly allocated to one of the two study arms (Sect. 2.1). We did not stratify the randomization given the comparable social and demographic characteristics of rural ASHAs in both arms (found in baseline assessment), such as age, education, and years of work experience, and unlike earlier trials in the region that involved ASHAs, ASHA Facilitators and Multi-Purpose Workers (Naslund et al., 2021), this study focused on a single cadre (ASHAs: rural, resident women) within a district. The data manager conducted the randomization using Stata statistical software version-14 (StataCorp, 2021).

ASHAs allocated to the intervention arm were informed separately (telephonically) by a member of the intervention team (who is unblinded) within a week from the date of baseline assessment, and the intervention team member called the ASHAs (batch-wise) and confirmed their availability to attend the residential workshop. All consented ASHAs (both arms) were invited by the members of the outcome evaluation team (blinded) for scheduled outcome (follow-up) assessments.

### Experimental Conditions

#### Regular Support Mechanisms (Control and Intervention Arms)

ASHAs typically receive regular support for their routine work-related problems through two means: a) facilitators; b) more experienced peer ASHAs.

ASHAs in this study were supported by their routine ‘ASHA Facilitators’. Facilitators typically supervise a group of ~ 20 ASHAs through face-to-face meetings. According to the description of their roles (National Health Mission Handbook for ASHA Facilitators and MPWs on HBNC and HBYC, n.d.), one of their critical tasks is to provide support to the ASHAs and ‘mentor’ them to improve their effectiveness, particularly for ASHAs who function poorly on many of their tasks, or remain absent from Village Health and Nutrition Days (VHNDs) or monthly meetings/trainings. ASHA Facilitators ensure that particularly these ASHAs receive extra mentoring support and encouragement from them. The handbook instructs that ASHA facilitators identify such ASHAs during village visits or cluster meetings, and thereafter, identify the reasons for their poor performance. Some possible reasons include ASHA’s low skill or knowledge levels, delays in payments, irregular medication supply for their services, inappropriate behaviors of the health system staff, lack of family support for working, illness in the family, other social barriers, or no interest in continuing as ASHAs. The aforementioned scope of support for ASHAs and easy access to facilitators (who are also village-based resident women) create spaces for ASHAs to discuss their work-stress issues with their facilitators and receive guidance on coping with stress-inducing situations.

Notably, ASHAs with substantial community experience are usually appointed (promoted) to the facilitator position, and in this respect, ASHAs also use opportunities to discuss/share their work stress issues or seek the support of their ‘older peers’, which has been reported in prior qualitative work in the same context (Shrivastava et al., 2023).

#### Regular Support Mechanisms and Character-Strengths based Coaching (Intervention Arm)

In addition to their regular support mechanisms, ASHAs in the intervention arm received a structured character-strengths based coaching intervention i.e., the initial 5-day residential workshop and follow-on 8- to 10-week (weekly) remote telephonic coaching support. Details of the development and content testing of this intervention have been discussed elsewhere (Khan et al., 2023).

ASHAs first received a residential workshop on using character strengths in their work, to respond to or cope with stressful situations. The workshop was delivered by two members of the intervention development team: the intervention development lead (IL) and the intervention coordinator (IC), with a couple of intervention coaches attending the sessions. We administered a pre- and post-workshop knowledge assessment test. The subsequent remote personalized telephonic coaching (weekly) by these intervention coaches (who had become familiar with the ASHAs by then), who were trained on the coaching strategies, was delivered for 8–10 weeks to batches of ASHAs who had participated in the workshop. Remote coaching used scalable and low-cost approaches, including once-a-week phone calls and WhatsApp support—the latter was used to share content on the use of character-strengths (reinforcing workshop learnings) and answer any contingent queries by ASHAs, in addition to reminding them of the scheduling of their successive coaching calls. Coaches also obtained verbal consent of ASHAs for recording their calls and a random sub-sample of these calls were listened to by the IL and IC, and study investigators for quality. We developed a checklist for rating ASHAs’ calls referring to items of therapist competence checklists used in similar contexts in India (Kohrt et al., 2015). Checklists were also self-rated by ASHAs, and fortnightly meetings convened by the IL and IC discussed these ratings with the coaches, to provide supportive feedback to the coaches.

The intervention development resulted in a self-directed learning-based booklet, ‘the content manual,’ for ASHAs consisting of four modules. The modules adhered to the intervention blueprint (Khan et al., 2023), and included topic-wise information on the understanding of character-strengths; nature of personal, workplace and community-level challenges faced by ASHAs; their effects on physical health and mental wellbeing; the ways to evaluate stress-inducing situations; and evidence-based stress-response strategies (Khan et al., 2023), in addition to reflective practice-exercises. The character-strengths based stress-response strategies have been detailed in a blueprint from our prior intervention development paper (Khan et al., 2023, refer supplemental files 1 and 2). The content manual was a reference for ASHAs during the workshop and later, while remote coaching iteratively addressed ASHAs’ evolving work-stress issues, as ASHAs resumed their routine work after the workshop.

Finally, we administered a structured 10-item feedback form (multiple choice questions) at the end of each of the three batches of workshops for ASHAs, to gather their experiences of the workshop and perspectives on the workshop content (e.g., its clarity and utility) and delivery. We used the feedback form data to conduct a manipulation check and examine if the experiences of the workshop had a relationship with wellbeing scores of ASHAs at 3-month follow-up.

### Outcome Assessment

We collected outcomes on preliminary effectiveness of the intervention and its acceptability and feasibility.

Study outcomes were collected at baseline, and thereafter at 1-month, 3-month and 6-months using paper-based forms. Primary outcome analysis was defined at 3-month follow-up based on earlier studies of positive psychological interventions using the Authentic Happiness Inventory (Proyer et al., 2017), and the roles of affect, self-efficacy, and perception of flourishing in affecting authentic happiness levels were assessed at 1-month follow-up. Secondary effects of the intervention on burnout and motivation were assessed at 3-month follow-up. Longer-term intervention effects on authentic happiness, in addition to any effects on burnout, motivation and self-reported quality of life were assessed at 6-month follow-up.

The average duration of an assessment visit was approximately 2–3 h. We provided compensation to ASHAs for the assessments (~ USD 6 per ASHA), sufficient breaks to answer the study questionnaires, and refreshments/snacks owing to the long assessment periods. Most assessments were self-filled by ASHAs and as needed, the outcome assessor (research assistant) provided support to administer the questions. Outcome assessors who were blind to arm allocation, and who were not involved in intervention development, collected the assessment data. Table 1 provides more details on individual outcome measures.

**Table 1:** Summary of study measures administered to ASHAs

#	Measure	Instrument with psychometric properties	#Items	Description	Time point
1	Subjective wellbeing	Authentic Happiness Inventory (AHI) Cronbach’s α ≥ .91; Comparative Fit Index (CFI) > 0.90 (Proyer, 2017)	24	AHI includes 24 sets of five statements [e.g., ranging from 1 (“I feel like a failure”) to 5 (“I feel I am extraordinarily successful”)]. The respondent chooses the statement that best describes her feelings in the past one week. Total score = 120	Baseline, 3 & 6 months
2	Affect	Positive and Negative Affect Schedule (PANAS) Short Form (I-PANAS-SF) Cronbach’s α = 0.78 and 0.76 respectively for PA and NA subscale; CFI = 0.94 (Thompson, 2007)	10	10-item scale to measure the ASHA affect; total score is calculated by finding the sum of 10 positive items and then 10 negative items. Score ranges from 10 to 50 for both sets of items. A higher total positive score and a lower total negative score, indicates improved mood	Baseline, 1 & 3 months
3	Flourishing	VanderWeele’s Index: ‘Flourish Index (FI)’ and ‘Secure Flourish Index (SFI)’ Cronbach’s α = 0.89 for FI and 0.86 for SFI; CFI = 0.97 for FI and SFI (Weziak-Bialowolska, 2019)	12	FI consists of two questions from each of the following five principal domains: happiness and life satisfaction, mental and physical health, meaning and purpose, character and virtue, and close social relationships. Each of the questions is assessed on a scale of 0–10. FI score is obtained by adding the scores from each of these ten questions (total: 0–100). SFI follows the same method, with the domain specific scores of the last two items	Baseline, 1 & 3 months
4	Self-efficacy	Occupational Self-Efficacy Scale (OSES) Cronbach’s α = 0.87; CFI > 0.90 (Chaudhary, 2014)	19	Includes six dimensions: confidence, command, adaptability, personal effectiveness, positive attitude and individuality. Each item scored on a 5-point Likert’s scale from ‘strongly disagree’ to ‘strongly agree’, with higher total scores reflecting higher levels of self-efficacy	Baseline, 1 & 3 months
5	Burnout	Maslach Burnout Inventory- Emotional Exhaustion (EE) subscale Cronbach’s α = 0.88 (Emotional Exhausion subscale); CFI = 0.88 (Pisanti et al. 2013)	9	EE subscale has 9 items, each with 7-point Likert-type. The EE score includes nine items with a range of 0–54 (low burnout: 0–16, moderate: 17–26, high: ≥ 27)	Baseline, 3 & 6 months
6	Motivation	Health worker motivation tool Cronbach’s α = 0.75; Three-factor structure explained > 90% of variance in data Mbindyo (2009)	23	The tool has eight major constructs: general motivation, burnout, job satisfaction, intrinsic job satisfaction, organizational commitment, conscientiousness, timeliness, and personal issues. Answers are given on an agreement scale of 1–4 (1 = strong disagreement, 4 = strong agreement). Maximum score: 92	Baseline, 3 & 6 months
7	Quality of Life	EQ-5D Cronbach’s α = 0.77; CFI = 0.99 Bilbao (2022)	5	One question for each of the five dimensions: mobility, self-care, usual activities, pain/discomfort, and anxiety/depression. The answers permit the tool to find 243 unique health states to convert into an index score anchored at 0 for death and 1 for perfect health. The Visual Analog Scale (VAS) helps report the perceived health status with ranging from 0 (worst possible health status) to 100 (best possible health status)	Baseline, 3 & 6 months
8	Character Strengths	Self-Perceived Strengths (SPS) Test–retest reliability of 0.43–0.8 and concurrent validity of 0.39–0.67 (Mehrotra, 2015)	24	Each vignette has a hypothetical individual having the characteristics of a character-strength, without specifying its name. Participants specify the degree of similarity-dissimilarity with this person (“very different from me” (1) to “very much like me” (6)). Total strength scores out of 120 are also calculated	Pre- and post-intervention (*intervention arm only*)

At baseline, ASHAs provided social and demographic information such as their age, level of education, years of experience, family type (joint/nuclear), number of children, number of training modules they completed (part of induction into the ASHA role) and population of their village (refer Table 2).

**Table 2:** Arm-wise socio-demographic data (collected at baseline from the participants)

	Control arm (N = 35)	Intervention arm (N = 35)
Average age (years)	34.94	38.14
Years of work experience	0–5 years: 8 6–10 years: 15 11–15 years: 4 16–20 years: 8 > 21 years: 0	0–5 years: 4 6–10 years: 16 11–15 years: 8 16–20 years: 7 > 21 years: 0
Family type	Joint: 21 Nuclear: 14	Joint: 21 Nuclear: 14
Number of induction training modules completed (ASHA training under National Health Mission)	6.2	6.3
Population of the ASHA’s assigned village	< 1000: 28 1000–2000: 5 > 2000: 2	< 1000: 25 1000–2000: 6 > 2000: 4
Number of children	0: 0 1: 7 2: 8 > 2: 20	0: 1 1: 5 2: 13 > 2:16
Education level	< Grade-10: 17 Grade 10: 7 Grade 11: 2 Grade 12: 6 Graduate: 3 Post graduate: 0	< Grade 10: 11 Grade 10: 16 Grade 11: 1 Grade 12: 4 Graduate: 2 Post graduate: 1

#### Preliminary Effectiveness of the Intervention

We have included details on reliability and validity of outcome assessment tools in Table 1. During the formative phase of the larger study (2021), the outcome assessment tools were forward translated from English to Hindi by a bilingual expert within the team (AK) to facilitate its use in Indian contexts. An independent bilingual team member performed the back-translation, and discrepancies were reconciled through consensus. Both the translators worked independently, and having fluency in both languages, and an awareness of cultural nuances, they have also performed translations earlier for similar studies involving psychometric tools in Sangath’s experience of twelve years in the study region. The evaluation of the back-translation focused on conceptual equivalence (Beaton et al., 2000). We conducted pilot testing of the tools with a small sample of ASHAs (n = 30) to ensure the translated tool’s clarity and understanding.

##### Primary Outcome

Authentic Happiness Inventory (AHI) (Seligman et al., 2005) is a frequently used instrument for the subjective assessment of happiness in positive psychology intervention studies (Proyer et al., 2017). We have used the terms happiness or (subjective) wellbeing in this paper, to represent this specific measure of happiness. AHI includes 24 sets of five statements [e.g., ranging from 1 (“I feel like a failure”) to 5 (“I feel I am extraordinarily successful”)], from which the respondent chooses the statement that best describes their feelings in the past one week. AHI has been designed for monitoring upward changes in happiness (Seligman et al., 2005) and has often been used in PPI studies (Mongrain & AnselmoMatthews, 2012; Proyer et al., 2014; Ruch et al., 2010; Schiffrin & Nelson, 2010). Internal consistency at pre-test has been reported to be high (Cronbach’s α = 0.94). AHI has been used previously in studies in India (Ruparel et al., 2022; Tiwari et al., 2013). AHI was administered at baseline, and at 3 and 6 months thereafter.

The AHI items show associations with other aspects of routine ‘wellbeing’ and specifically, in comparison to instruments assessing the satisfaction with life, the AHI has shown stronger relationships to other wellbeing indicators such as the orientations to happiness, depressive symptoms, and emotions such as hope, interest, joy, or pride (Proyer et al., 2017). The AHI items fit well into Seligman’s conceptualization of happiness (Seligman, 2002) and to a large extent, his conceptualization of flourishing (Seligman, 2011). Therefore, assessing AHI scores is expected to provide a meaningful picture of the ASHA’s ‘current’ levels of subjective happiness/wellbeing, and its associated benefits in day-to-day life.

##### Secondary Outcomes

We included the secondary outcome measures of affect, flourishing and self-efficacy to assess their possible roles in affecting wellbeing/authentic happiness at 3-month follow-up. The secondary outcome measures also included an assessment of the more extrinsic effects of changes in authentic happiness/wellbeing, for which we used the emotional exhaustion subscale of the Maslach Burnout Inventory—Human Service Survey, and the motivation scale, used in earlier research for Indian frontline health workers. We also assessed ASHA’s self-reported quality of life to examine potential correlations between perceived quality of life and wellbeing. Finally, we measured self-perceived character strengths of ASHAs (exploratory outcome) in the intervention arm at baseline and after the last remote coaching call with the underlying assumption that an ASHA’s overall self-perception of character strengths would increase after the full receipt of the intervention.

For assessing affect, we used the 20-item International Positive and Negative Affect Schedule (PANAS) Short Form (I-PANAS-SF) (Thompson, 2007). The ‘Flourish index’ (VanderWeele, 2017) was used to assess flourishing, including two questions from each of the five principal domains of happiness and life satisfaction, mental and physical health, meaning and purpose, character and virtue, and close social relationships. The Occupational Self Efficacy Scale (OSES) (Chaudhary, 2014) was used to assess self-efficacy. The emotional exhaustion sub-scale of the Maslach Burnout Inventory–Human Service Survey (Maslach & Jackson, 1986, 2012; Pisanti et al., 2013) was used to assess burnout in terms of emotional exhaustion, given the emotional labour experienced by ASHAs (Pandey & Singh, 2015). The motivation scale pretested for Indian frontline health workers (Tripathy et al., 2016; Mbindyo, 2009) was used to assess motivation levels. The instruments for assessing affect, self-efficacy and flourishing were administered at baseline, 1- and 3-months, and extrinsic effects were assessed at baseline, 3- and 6-months. The self-perceived character-strengths of ASHAs (Mehrotra et al., 2015; Tripathi et al., 2015) of the intervention arm were assessed at baseline, before the intervention workshop, and after completion of the last remote coaching call. Perceived quality of life and emotional wellbeing of ASHAs was assessed using the EQ-5D scale with the visual analogue scale (Bilbao et al., 2022; Hinz et al., 2013; Jyani et al., 2023), with one question for each of the five dimensions that included mobility, self-care, usual activities, pain/discomfort, and anxiety/depression.

#### Feasibility and Acceptability of the Intervention

##### Qualitative Data

Two research assistants conducted a total of 12 interview phone-calls with 12 out of 29 ASHAs who attended the residential workshop and were engaged with remote coaching calls. An interview guide with semi-structured questions was prepared to assess an ASHA’s perspective/experience/feedback on remote telephonic coaching. The telephone interviews focused on ASHAs’ experiences of the remote coaching component, as their experiences of the workshop were already explored during the intervention content testing phase (Khan et al., 2023). A research assistant commenced the interview call after taking the ASHA’s verbal consent; the call was not recorded, but detailed written notes were documented by another research assistant. Average time taken to complete an interview was ~ 27 min.

The written notes were documented into fair notes in English (MS Word) for each ASHA and this process helped the coder in getting familiarized with the data (Braun & Clarke, 2006). A single coder (SA) started the initial coding of the data using MS Excel 2010, and the codes and sub-codes were finalized after discussion with the study co-investigator (APB) (more details in 2.9.2). A second coder, post hoc, coded the same data to calculate the inter-coder agreement (Cohen’s kappa).

##### Quantitative Data

We collected intervention process indicators to determine ASHAs’ engagement and use of each of the components of the intervention i.e., residential workshop and remote coaching. These indicators included: (1) number of ASHAs who attended the 5-day residential workshop out of those who were called/invited, (2) number of ASHAs with whom the first remote telephonic call was scheduled out of those who completed the workshop, (3) number of ASHAs who completed all eight weekly telephonic calls, (4) average coaching call duration, and (5) number of ASHAs who dropped out between workshop commencement and their last coaching call.

### Data Analysis

#### Preliminary Effectiveness of the Intervention

The primary effectiveness analysis was intention-to-treat. The main statistical analysis estimated the difference in the total authentic happiness score (as group mean) between ASHAs randomized to the coaching intervention (intervention arm) versus routine supervision (control arm) at 3-month follow-up. To estimate this difference, we used unpaired t test and ANCOVA with repeated measures. These total authentic happiness scores were also compared at baseline to check for between-arm comparability, and at 6-month follow-up to assess any potential longer-term effect of the intervention. We reported unadjusted and baseline-adjusted differences in total scores with 95% confidence intervals. All analyses were completed using Stata (StataCorp, 2021) and *p *< 0.05 was considered statistically significant.

As part of exploratory analysis, we also compared the scores on secondary outcome measures of burnout and motivation; affect, self-efficacy and flourishing, and perceived quality of life and emotional wellbeing, between the arms at baseline, 1-month, 3-month and 6-month follow-up, as applicable to the measure (see Sect. 2.8.1.2). Finally, we examined before- and after-intervention scores to explore any changes in self-reported character-strengths of ASHAs of the intervention arm.

#### Feasibility and Acceptability of the Intervention

Quantitative process indicators to assess intervention feasibility and acceptability were analyzed descriptively, such as proportion of ASHAs inducted into the intervention and proportion of ASHAs who received the optimum intervention dose (Sect. 2.8.2.2.).

With respect to telephonic interviews with intervention arm ASHAs aiming to gather their perceptions and experiences of remote coaching, thematic analysis (Braun & Clarke, 2006) flexibly allowed us to include both a priori or ‘deductive,’ pre-existing themes from the interview guide, as well as themes that emerged during the analysis or ‘inductive’ themes. SA independently coded the data, reflecting important sections of the interview guide, and with APB, refined the codes using deductive-inductive approaches. Through consultation calls between SA and APB, subsequent iterations were made to the codes i.e., by adding new codes, deleting redundant codes, and integrating the overlapping codes, and disagreements on the classification of themes and subthemes were resolved. Inter-rater agreement was calculated to assess coding consistency between the two researchers who coded the data i.e., the first researcher (SA) coded the data during the pilot (2022–23) and a second researcher coded the same data post hoc in 2024. After independently coding all of the data, inter-rater agreement (Halpin, 2024) was calculated by an independent researcher who was not involved in the coding and we obtained an inter-rater agreement (Cohen’s kappa) of 0.81.

The themes and subthemes were compiled with inclusion of salient ASHA quotes, and further reviewed, refined, and named for the final report (Braun & Clarke, 2006). Themes were broadly divided into remote coaching call ‘logistics’ (Table 10) and ‘experiences’ (Table 11) where logistics included ASHAs’ perspectives on the operations of the calls (e.g., scheduling, challenges in attendance, duration, frequency, or reminders) and experiences included their perspectives on the calls themselves (e.g., perceived connection with the coach, or knowledge gained, or reported applications of coaching strategies, or use of character-strengths).

#### Manipulation Check

We used the scores obtained by ASHAs on the 10-item feedback form (minimum score: 4; maximum score: 33) discussed in Sect. 2.7.2 to assess their experience and perspective of the workshop content’s clarity and utility, and their degree of satisfaction with the workshop facilitator’s (AK) delivery of the workshop, and the staying/food/other arrangements at the workshop venue. We analyzed this data overall in terms of mean feedback scores, and also used Pearson correlation coefficients to examine the relationship between feedback scores for the workshop and the change in wellbeing scores from baseline to 3-month follow-up.

## Results

### Preliminary Effectiveness Results

Participant characteristics are summarized in Table 2. We observed comparability in the distribution of social and demographic characteristics (such as age, education level, and years of experience) between arms (Table 2). Average total scores at baseline, 3 month and 6-month follow-up are provided in Table 3.

**Table 3:** Average total scores at baseline, 3 months and 6 months

Measure	Baseline	3-month follow-up	6-month follow-up
	Average of total score (standard deviation)		
Authentic happiness inventory (AHI)	80.1 (13.9)	79.9 (13.63)	81.85 (14.81)
Emotional exhaustion subscale of maslach burnout inventory	24.94 (9.17)	23.92 (10.59)	22.98 (9.43)
Motivation scale	71.9 (7.9)	73.4 (6.5)	71.5 (6.7)
Positive affect (PANAS)	27.4 (4.83)	28.40 (5.28)	29.41 (4.94)
Negative affect (PANAS)	28.2 (6.80)	27.37 (5.96)	27.62 (5.43)
Occupational self-efficacy scale	77.8 (11.7)	78.77 (7.92)	78.11 (9.84)
Flourish index	7.53 (1.36)	8.06 (1.13)	7.86 (1.20)
Secure flourish index	7.26 (1.34)	7.79 (1.13)	7.67 (1.18)
EQ-5D utility index	0.90 (0.12)	0.84 (0.18)	0.89 (0.09)
Visual analogue scale	82.21 (18.09)	80.49 (17.55)	82.37 (16.04)

Using unpaired two-sample t tests (Table 4), we explored if there were statistically significant mean differences between arms at 3-month and 6-month follow-up for the primary outcome measure (subjective wellbeing/authentic happiness) and secondary measures (e.g., burnout and motivation). We also descriptively explored the roles of affect, flourishing and self-efficacy (if any) in the relationship between receiving the intervention and authentic happiness at 3-month follow-up. Finally, we examined the ASHAs’ self-reported quality of life at baseline, 3-month and 6-month follow-up, and self-perceived strengths scores (intervention arm) before the workshop and after the last remote coaching call. We have reported Cohen’s d as a measure of effect size (Table 4), ANCOVA to assess between-arm differences in scores adjusted for baseline scores (Table 5), and repeated measures ANCOVA to see any effect of time elapsed between follow-ups adjusting for arm and baseline scores (Table 6). For quality-of-life index, VAS and self-perceived strengths score, we conducted correlations to discuss the interpretable relationships between these variables and AHI (Table 7), emotional exhaustion and motivation scores (Tables 8 and 9).

**Table 4:** Average total scores at baseline, 3 months and 6 months (as applicable for the measure) and effect sizes, by arm

Arm	Mean of total score (standard deviation)				Magnitude of Cohen’s d (95% CI)		
	Baseline	1-month	3-month	6-month	1-month	3-month	6-month
*Authentic happiness inventory (AHI)*							
Control	82.6 (14.1)		76.32 (13.16)	80.67 (16.33)		0.437 (− 0.067, 0.941)	0.124 (− 0.382, 0.631)
Intervention	77.7 (13.4)		83.6^*a*^ (13.32)	83.03 (13.23)		0.29 (− 0.219, 0.799)	0.198 (− 0.318, 0.714)
*Emotional exhaustion subscale of maslach burnout inventory*							
Control	23.8 (8.6)		24.8 (11.1)	22.5 (9.7)		0.02 (− 0.478, 0.518)	0.251 (− 0.257, 0.759)
Intervention	26.0 (9.7)		23.0(10.2)	23.5 (9.3)		0.142 (− 0.365, 0.649)	0.124 (− 0.391, 0.639)
*Motivation scale*							
Control	72.0 (8.5)		74.2 (7.1)	70.7 (7.8)		0.253 (− 0.247, 0.752)	0.236 (− 0.272, 0.744)
Intervention	71.9 (7.4)		72.5 (5.9)	72.2 (5.3)		0.077 (− 0.430, 0.583)	0.201 (− 0.315, 0.717)
*Positive affect (PANAS)*							
Control	26.71 (4.90)	27.93 (4.78)	28.94 (4.65)		0.235 (− 0.250, 0.719)	0.409 (− 0.094, 0.912)	
Intervention	28.09 (4.72)	28.93 (5.84)	29.9 (5.26)		0.101 (− 0.414, 0.617)	0.342 (− 0.167, 0.852)	
*Negative affect (PANAS)*							
Control	27.08 (6.4)	26.54 (5.71)	27.90 (5.81)		0.119 (− 0.364, 0.602)	0.077 (− 0.421, 0.575)	
Intervention	29.31 (7.06)	28.31 (6.19)	27.33^c^ (5.09)		0.348 (− 0.170, 0.867)	0.481 (− 0.032, 0.995)	
*Occupational self-efficacy scale*							
Control	77.49 (13.18)	78.27 (8.35)	79.94 (7.92)		0.066 (− 0.416, 0.549)	0.136(− 0.363, 0.634)	
Intervention	78.11 (10.19)	79.34 (7.51)	76.23 (11.32)		0.028 (− 0.487, 0.543)	0.214 (− 0.294, 0.721)	
*Flourish index*							
Control	7.49 (1.48)	8.18^b^ (1.02)	7.98^c^ (1.24)		0.647 (0.152, 1.142)	0.444 (− 0.060, 0.948)	
Intervention	7.57 (1.24)	7.92 (1.25)	7.73 (1.17)		0.271 (− 0.246, 0.788)	0.134 (− 0.373, 0.640)	
*Secure flourish index*							
Control	7.13 (1.37)	7.85^b^ (1.07)	7.74^c^ (1.23)		0.673 (0.177, 1.169)	0.538 (0.031, 1.045)	
Intervention	7.38 (1.31)	7.74 (1.23)	7.60 (1.14)		0.276 (− 0.241, 0.794)	0.187 (− 0.320, 0.694)	
*EQ-5D utility index*							
Control	0.92 (0.13)		0.84^c^ (0.19)	0.89^d^ (0.08)		0.677 (0.156, 1.197)	0.599(0.073, 1.125)
Intervention	0.89 (0.12)		0.84 (0.17)	0.88 (0.11)		0.44 (− 0.072, 0.952)	0.182 (− 0.334, 0.698)
*Visual analogue scale (VAS)*							
Control	86.29 (15.21)		82.42 (14.60)	85 (14)		0.304 (− 0.197, 0.805)	0.068 (− 0.438, 0.575)
Intervention	78.14 (19.97)		78.5 (20.22)	79.7 (17.8)		0.062 (− 0.444, 0.568)	0.039(− 0.475, 0.554)

^a^Cohen’s d = 0.55 (95% CI 0.04, 1.06) for between-arm difference at 3-month follow-up

^b^*p* < 0.05—significant between baseline to 1-month follow-up

^c^*p* < 0.05—significant between baseline to 3-month follow-up

^d^*p* < 0.05—significant between baseline to 6-month follow-up

**Table 5:** Analysis of Co-variance (ANCOVA) results by measure for between-arm difference in mean total scores, adjusted for baseline score, at 1, 3 and 6-month follow-up (as applicable for the measure)

Measure and follow-up time-point	Sum of Square	Degree of Freedom	Mean Sum of Square	F-statistic	*p* value
AHI- 3 month	961.36013	1	961.36013	5.86	**0.0187**
AHI- 6 month	179.10234	1	179.10234	1.06	0.3083
Emotional exhaustion- 3-month	36.22177	1	36.22177	0.37	0.5459
Emotional exhaustion- 6-month	16.137444	1	16.137444	0.2	0.6589
Motivation- 3-month	57.991297	1	57.991297	1.63	0.2062
Motivation 6-month	23.198356	1	23.198356	0.59	0.4449
Positive affect- 1-month	3.7	1	3.7	0.14	0.709
Positive affect-3-month	5.0	1	5.0	0.22	0.645
Negative affect-1-month	8.8	1	8.8	0.27	0.602
Negative affect-3-month	44.6	1	44.6	2.01	0.1612
Self-efficacy-1-month	13.2	1	13.2	0.22	0.640
Self-efficacy-3-month	206.9	1	206.9	2.67	0.1075
Flourish index-1-month	1.4	1	1.4	1.12	0.295
Flourish index-3-month	1.7	1	1.7	1.42	0.2383
Secure flourish index-1-month	0.5	1	0.5	0.43	0.515
Secure flourish index-3-month	1.2	1	1.2	1.07	0.3042
EQ-5D-3-month	0.002	1	0.002	0.07	0.7857
EQ-5D-6-month	0.00002	1	0.00002	0	0.959
VAS-3-month	58.0	1	58.0	0.2	0.660
VAS-6-month	164.2	1	164.2	0.69	0.410

Detailed results are included in the supplemental tables

**Table 6:** Repeated measures ANCOVA (within individuals), by measure

	Source of variation	Co-efficient	Standard Error	z statistic	*p* value	95% CI UL	95% CI LL
AHI	Time	1.9	1.9	1.0	0.297	− 1.7	5.6
	Arm	5.7	2.7	2.1	0.036	0.4	11.1
Emotional exhaustion subscale	Time	− 0.8	1.2	− 0.7	0.504	− 3.1	1.5
	Arm	− 0.4	2.1	− 0.2	0.865	− 4.4	3.7
Motivation scale	Time	− 2.1	0.9	− 2.2	0.025	− 3.9	− 0.3
	Arm	− 0.5	1.2	− 0.4	0.666	− 2.9	1.9
Positive affect (PANAS)	Time	0.9	0.7	1.2	0.226	− 0.5	2.3
	Arm	0.6	1.0	0.6	0.537	− 1.4	2.6
Negative affect (PANAS)	Time	0.3	0.7	0.4	0.707	− 1.1	1.6
	Arm	− 0.5	1.1	− 0.4	0.686	− 2.7	1.8
Self-efficacy	Time	− 0.5	1.4	− 0.3	0.748	− 3.2	2.3
	Arm	− 1.2	1.5	− 0.8	0.435	− 4.2	1.8
Flourish index	Time	− 0.2	0.2	− 1.3	0.194	− 0.5	0.1
	Arm	− 0.3	0.2	− 1.4	0.171	− 0.8	0.1
Secure flourish index	Time	− 0.1	0.1	− 0.8	0.402	− 0.4	0.2
	Arm	− 0.2	0.2	− 1.0	0.311	− 0.7	0.2
EQ-5D utility index	Time	0.05	0.02	2.08	0.038	0.003	0.09
	Arm	0.01	0.03	0.20	0.841	− 0.05	0.06
VAS	Time	1.9	2.4	0.8	0.435	− 2.8	6.5
	Arm	− 2.9	3.4	− 0.8	0.402	− 9.6	3.9

**Table 7:** Average of total strength scores and individual strength scores on self-perceived strengths assessment (intervention arm) before the intervention workshop (pre-intervention) and after the last remote coaching call (post-intervention)

	Character strengths	Pre-intervention mean score (SD)	Post- intervention mean score (SD)	Difference of means
1	Leadership	4.2(1.4)	4.6(1.5)	− 0.4
2	Forgiveness	3.8(1.5)	4.9(1.2)	− 1.1*
3	Appreciation of beauty	4.0(1.6)	4.5(1.6)	− 0.5
4	Justice and fairness	4.2(1.3)	4.5(1.3)	− 0.3
5	Spirituality	3.5(1.6)	4.4(1.5)	− 0.9*
6	Social skilfulness	4.0(1.6)	4.7(1.2)	− 0.7
7	Hope	4.3(1.5)	4.8(1.1)	− 0.5
8	Sense of humour	4.1(1.6)	4.3(1.3)	− 0.2
9	Social responsibility	3.6(1.5)	4.5(1.4)	− 0.9*
10	Courage	4.1(1.2)	4.6(1.1)	− 0.5
11	Kindness	4.3(1.4)	4.7(1.4)	− 0.4
12	Persistence	4.1(1.3)	4.5(1.3)	− 0.4
13	Close and loving relationships	3.4(1.7)	4.2(1.3)	− 0.8*
14	Genuineness and honesty	3.8(1.8)	4.6(1.4)	− 0.8*
15	Practical and farsightedness	4.2(1.5)	4.5(1.3)	− 0.3
16	Creativity	3.1(1.4)	3.9(1.4)	− 0.8*
17	Modesty and humbleness	3.8(1.6)	4.2(1.2)	− 0.4
18	Curiosity	3.4(1.6)	4.0(1.6)	− 0.6
19	Self-discipline	3.8(1.5)	4.3(1.3)	− 0.5
20	Wisdom	3.6(1.7)	4.6(1.2)	− 1*
21	Being energetic and lively	3.9(1.6)	3.8(1.4)	0.1
22	Open mindedness	3.6(1.3)	4.1(1.0)	− 0.5*
23	Gratitude and thankfulness	4.1(1.5)	4.7(1.4)	− 0.6
24	Love to learn new things	4.8(1.4)	4.5(1.1)	0.3
	**TOTAL STRENGTH SCORE**	**93.6(17.0)**	**106.4(18.8)**	12.8*

^*^p < 0.05

**Table 8:** Pearson correlation (with significance testing) between EQ-5D utility index and VAS scores, and AHI, emotional exhaustion and motivation scores between arms at baseline and follow-up assessments

Measure	Correlation with EQ-5D (p value)				Correlation with VAS (p value)		
	Arm	Baseline	3-month	6-month	Baseline	3-month	6-month
AHI	Control	0.07 (0.69)	− 0.19 (0.28)	− 0.17 (0.36)	− 0.29 (0.08)	0.30 (0.09)	0.20 (0.27)
	Intervention	0.28 (0.09)	0.44 (0.01)	0.16 (0.37)	− 0.04 (0.78)	0.36 (0.04)	0.11 (0.54)
Emotional exhaustion subscale	Control	− 0.00 (0.99)	− 0.43 (0.01)	− 0.26 (0.16)	0.04 (0.78)	0.05 (0.75)	− 0.16 (0.38)
	Intervention	− 0.28 (0.09)	− 0.57 (0.00)	− 0.54 (0.00)	− 0.30 (0.07)	− 0.17 (0.36)	− 0.39 (0.03)
Motivation	Control	0.09 (0.6)	0.07 (0.68)	0.13 (0.48)	0.12 (0.47)	0.13 (0.46)	0.09 (0.60)
	Intervention	− 0.05 (0.75)	0.49 (0.00)	− 0.17 (0.35)	− 0.06 (0.69)	0.44 (0.01)	− 0.01 (0.95)

**Table 9:** Pearson correlation (with significance testing) between self-perceived strengths scores, and AHI, emotional exhaustion and motivation scores in the intervention arm, at baseline and follow-up assessments

Measure	Correlation with SPS score (p value)	
	Baseline	3-month
AHI	0.13 (0.50)	0.14 (0.49)
Emotional exhaustion	− 0.15 (0.43)	− 0.07 (0.73)
Motivation	0.47 (0.01)	0.31 (0.13)
EQ-5D	0.05 (0.79)	0.00 (0.99)
VAS	0.28 (0.13)	0.02 (0.91)

At 3-month follow-up, we found that ASHAs in the intervention arm scored higher means of total scores of subjective wellbeing (Table 4) on the Authentic Happiness Inventory (n = 30, Mean = 83.6; SD = 13.32) compared to ASHAs in the control arm (n = 31, Mean = 76.32; SD = 13.16), with a maximum attainable score of 120. This represents a significant difference of 7.28 points (t = 2.146, 95% CI 0.49 to 14.06, *p* = 0.0359). Table 4 also shows that the mean total scores in the intervention arm were nearly unchanged (n = 28, Mean = 83.03, SD = 13.23) at 6-month follow-up, relative to 3-month follow-up; however, among the control arm ASHAs, the scores were still lower relative to baseline (n = 31, Mean = 80.67, SD = 16.33). The between-arm difference at 6-month follow-up in total AHI scores was non-significant. ASHAs in the control arm showed a decline in scores between baseline and 3-month follow-up, followed by an increase between 3-month and 6-month follow-up but without attaining baseline score levels. The between-arm difference in average of total AHI scores at 3-month follow-up corresponded to a Cohen’s d effect size (Table 4) of 0.55 (95% CI 0.04–1.06). Note that we had hypothesized an effect size of 0.69 in the power analysis (Sect. 2.3). ANCOVA results indicate that the between-arm difference at 3-months (and not at 6-months) was significant adjusting for baseline scores (Table 5). Further, the time elapsed between 3 and 6-month follow-up (Table 6) is not affecting the score at 6-month follow-up (*p* = 0.297 for time) in either arm. Notably, the 3-month AHI scores are significantly correlated (Table 8) with EQ-5D and VAS scores in the intervention arm, but this needs to be interpreted with caution due to non-significant between-arm differences in EQ-5D and VAS scores at 3-month follow-up.

With respect to secondary outcome measures, both mean (of the total) emotional exhaustion scores and motivation scores (Table 4) did not show significant between-arm differences at 3-month or 6-month follow-up. The emotional exhaustion scores decreased between baseline and 3-month follow-up by three points in the intervention arm with a small effect size of 0.142 (− 0.365, 0.649). The emotional exhaustion scores at 3-month follow-up are significantly correlated with EQ-5D utility index scores for both arms (Table 8), possibly indicating that quality of life influenced the perceived emotional exhaustion at these time-points.

With reference to the assessments at 1-month and 3-month follow-up, and the descriptive role of affect in both arms, the positive affect scores marginally improved (i.e., increase in scores) from baseline to 3-month follow-up. Further, the between-arm differences at 1-month and 3-month follow-up for positive affect were non-significant. Negative affect scores showed a marginal increase (0.8 points) in the control arm and a decrease (~ 2 points) in the intervention arm from baseline to 3-month follow-up and between-arm differences were non-significant, as for positive affect. The self-efficacy scores showed a (non-significant) increase in the control arm from baseline to 3-month follow-up, but a (non-significant) decrease in the intervention arm, with non-significant between-arm differences in scores at 1- and 3-month follow-up.

The flourishing scores (FI and SFI scores, Table 4) showed an increase in both arms from baseline to 3-month follow-up, with non-significant between-arm differences at both follow-ups. The group means of the EQ-5D-5L quality of life index scores and visual analogue scale (VAS) scores also showed non-significant between arm differences at 3 and 6-month follow-up.

From the standpoint of self-perceived character-strengths expression (Table 7) among intervention arm ASHAs (exploratory outcome), the total strengths score significantly improved from 93.6 (pre-workshop) to 106.4 (post-last remote coaching call) and eight individual strengths (out of 24) also showed significant pre- and post-intervention improvements: forgiveness, spirituality, social responsibility, close and loving relationships, genuineness and honesty, creativity, wisdom, and open-mindedness.

### Intervention Feasibility and Acceptability Results

#### Quantitative Process Measures

Out of 35 ASHAs who were invited, 29 attended the 5-day residential workshop, who on average received a 16% higher post-workshop knowledge assessment score, compared to the pre-workshop score. Out of these 29 ASHAs, the first remote telephonic call was scheduled with 27 ASHAs. Out of these 27 ASHAs, 24 ASHAs received at least eight weekly telephonic coaching support calls (intended dose). About 221 calls were made to 24 ASHAs in total who received ≥ 8 calls, with an average call duration of 30.8 min. Five ASHAs (out of 29 who attended the workshop) dropped out between the workshop and the last scheduled coaching call.

#### Qualitative Findings

ASHAs who were telephonically interviewed (n = 12) for their feedback/perspectives on the experiences of remote coaching were ~ 37 years of age on average (27–45 years). In this group, 9 ASHAs had work experience of 6–10 years, 2 ASHAs had > 10 years of experience and 1 ASHA had < 5 years of experience. ASHAs showed a wide range of education levels: educated till 5th grade (n = 1), 8th grade (n = 1), 10th grade (n = 6), 12th grade (n = 3) and post-graduation (n = 1).

Results of analysis (themes and subthemes with salient quotes) were divided into two broad areas, remote coaching call ‘logistics’ (Table 10) and ‘experiences’ (Table 11). Remote coaching call *‘logistics’* includes the themes that pertain to the scheduling aspects of calls (‘Scheduling and rescheduling’), challenges faced while attending the remote coaching calls (‘Attending the call,’ including perspectives on domestic factors, time constraints, network connectivity and call privacy) and perspectives of ASHAs on call duration, call frequency and interval, and modes of call reminders. Remote coaching call *‘experiences’* covers the themes related to the experiences and learnings of the ASHAs during or after receiving the coaching calls; for e.g., perspectives of ASHAs on call content (content discussed during the call, the content manual, and content/reinforcing intervention messages shared through WhatsApp); the ways in which ASHAs connected with the coach; their experiences of remote coaching; retention of knowledge related to character-strengths or strategies based on character-strengths; and application of this knowledge in addressing work-stress. For more details on the themes and sub-themes as well as representative quotes of ASHAs, please refer to Tables 10 and 11.

**Table 10:** Themes and subthemes under coaching call ‘logistics’

Theme	Sub-theme	Sub-theme explanation	Quotes
Scheduling and rescheduling	Call scheduling	ASHA’s time is being considered in the scheduling, so ASHA finds it convenient. In one instance, an ASHA seemed compliant to the coach’s specified time	“Coach asks for the timing and when I have time, I tell it according to me.”
	Call rescheduling	Instances of rescheduling by ASHAs were less frequent over a period of 5 months of remote coaching (on average, twice a month for each ASHA). Possible reasons for rescheduling were time constraints due to personal and official work; health-related issues; network issues; and personal commitments (e.g., guests at home). There were a few instances where ASHA did not want to reschedule thinking that the coach may perceive that she (ASHA) has less interest	“If I am not able to talk then, we (ASHA & coach) find another time to talk.” “One time I was talking (coaching call), then the guests came, so I told (to coach) that we will talk next time.”
Attending the call	Domestic factors, constraints on time	Domestic factors affect ASHA’s call attendance, for example, housework, distraction because of family members and the possibility of her phone being used by somebody else (e.g., her child). ASHA may also be multi-tasking while she attends the call, which reflects her hectic schedule	“All the kids come around (near her); family members ask me what am I doing by sitting (in one place), when I talk to the coach.” “Sometimes I don’t get the time when there are many chores to do; yesterday, I also had headache, but it is necessary to talk (to the coach), to tell what I have done in the last week.”
	Network connectivity	Most of the ASHAs have no issues in terms of network connectivity while attending the calls. Some ASHAs expressed these issues in case of travelling and during rainfall (which shows that the coaching team can reschedule the call in such instances). They also expressed that they could manage with bad networks	“Never happened (network related issues). If it happened, then I will cut the phone and call again.” “Sometimes it (network issue) happens, the sound does not come, then I used to go to the terrace to talk.”
	Call privacy	Most ASHAs, recognizing that the topic of discussion could be about their work-stress, try to attend the call in a silent place, for example, a farm, roof/terrace, or a quiet place in the house. ASHAs recognize that talking in a crowded place can be hesitating (because of discussion on their work-stress narratives)	“Mostly at home, I attend the calls in a silent place” “I talked outside as well—when people pass by, I feel hesitant in talking.”
Call duration	Feedback on call duration	Most ASHAs are interested in having the call for at least half an hour (Average call duration: 30–35 min) and seem comfortable with calls up to 45 min to 1 h. However, they recognise their time constraints and hesitate to agree for a potentially longer call duration (seeing the perceived benefit/learning). Some ASHAs seem to be invested in the call and don’t realize the passing of an hour. At the same time, some ASHAs on occasions cannot be present for the average duration (30–45 min) due to work constraints	“I will feel good, if the call duration is increased because I will be learning.” “If the call duration is increased, it can be increased up to 1 h, but not more than that, because I (ASHA) am mostly busy.”
Call frequency and interval	Perspectives on between-call interval	Most ASHAs are compatible with an interval of 7–10 days between calls. However, there are sub-groups of ASHAs which have different perspectives: 1) Some ASHAs would want a shorter call interval because they have perceived good feelings about the conversation with the coach 2) Some ASHAs are indifferent to the interval because of their schedule 3) Some ASHAs are comfortable with a longer interval because they would then have more work-stress narratives to share with the coach	“Seven days gap was right. If the days will be less (difference between two calls), then it will be better. Getting to talk with the coach helps my day go well.” “Do it in 7 days or 10 days, there is no difference; what difference will 2 days make? I don’t have (that much) time.” “Ten days difference is good—in domestic life, something or the other keeps happening, so if the call happens in 15 days, then there will be more to talk.”
	Perspectives on call frequency	Most ASHAs are interested in attending more than the current number of calls (i.e., 8) possibly because of the positive perceptions about the call (coach checking on their well-being). With some ASHAs, their preference for number of calls will depend on their available time and yet with other ASHAs, they are okay with the current number of calls i.e., they would not want to increase the calls to more than eight	“Number of calls can be increased—I tried to learn (during the coaching), the training was beneficial. Eight calls are less, if the number of calls are increased, then it will be good.” “If you increase the number of calls, then also I will do. On occasions, time is not there, then it is a problem—and if time is there, then I can talk!”
Reminders	Feedback on coaching call reminder	Most ASHAs are comfortable with the current format of the coach reminding her over a telephonic call, and there is a preference for a call reminder versus reminder through texts (e.g., challenges with reading texts). Some ASHAs did prefer a text reminder over a call reminder. Some ASHAs are in the habit of putting reminders for themselves such as WhatsApp messages or calendar ticks. In terms of the ways of reminding them, ASHAs seem to be comfortable with a single reminder (for example, a day before, or an hour before—so that she can charge her phone). Only some ASHAs expressed the possibility of multiple reminders so that they won’t miss the call	“I am not able to see the messages because I am not much educated. My son has the phone, but he is also not that educated, that’s why reminder call is better than messages.” “I remember the timing of the call by putting the tick on the calendar.” “I used to tell that agricultural work is there, so I used to give the time of 12 o’clock noon, then coach does a reminder call at 11:00 am.”

**Table 11:** Themes and subthemes under coaching call ‘experiences’

Theme	Sub-theme	Sub-theme explanation	Quotes
Content (happenings) of Remote coaching Connection with the Coach	Content	A coaching call typically includes a conversation between a coach and an ASHA on a wide range of topics like character strengths (CS) and their usage; strategies (e.g., mindfulness or ‘three funny things’) and their application; and challenging routine life situations at work as well as at home	“When I come to home, then there is only work and work; I have shared about my work (related stress) with the coach. Coach has talked about making a list (prioritisation) if the work is more. I found it helpful but because when I get busy in work, I am not able to do it (properly).”
	Content manual	Most ASHAs did not open the content manual during the coaching call due to various reasons like not having a fixed place for attending the call, perception of no need of content manual or busy schedule. Some of them also expressed that opening the content manual will be helpful during the call. Some ASHAs opened the content manual during the call as well as afterwards and felt good after reading it	“Sometimes reading the book (content manual) also makes me feel good.” “During the call, I did not feel the need to open the content manual.” (*Note: The protocol does not state that the manual be used in the call; it is encouraged that the coach delivers the strategy after listening to the ASHA’s narrative and engages her in the conversation, rather than read out a strategy from the manual*)
	WhatsApp content	Most ASHAs received coaching call reminders through WhatsApp messages, but few ASHAs also received information regarding strategies. ASHAs (who did not receive WhatsApp content) expressed that it will be helpful if the information regarding strategies and CS will be shared through messages; except for one ASHA who was not able to read messages easily as she had difficulties in reading	“No information other than reminder calls is shared. Yes, it will be helpful, if information (strategies) is shared through WhatsApp or explained through video call.” (*Note: The protocol required regular sharing of static content on strategies to reduce work-stress [as explained in the workshop], over WhatsApp*)
	Ways of connection	ASHAs perceive a connection with the coach, which they have expressed through a range of opinions. For most ASHAs, it is a perception of ‘comfort’ (e.g., open to talk, coach is friend/sister-like, feeling of a bond, less hesitation or gradual loss of hesitation, ‘feeling good’ after talking to the coach, and feeling ‘heard’). In some instances, ASHAs have felt ‘unburdened’ (e.g., loss of tension/problems appearing less severe). In other instances, ASHAs have anticipated the coach’s call and felt motivated towards the calling. ASHAs also feel a connection, in part due to the coach’s attributes (e.g., the coach is perceived as a mentor; confidant; support or guide; or a good communicator), or the coach’s methods (e.g., way of explanation, use of examples, revision of workshop topics). A few ASHAs have critiqued the coach’s abilities (e.g., difficult to comprehend what they say). Some ASHAs have indicated the effects of power differences between them and an ‘external’ coach (e.g., hesitation in ‘saying something wrong’, or sharing personal problems may burden the coach, or feeling less able to give suggestions to the coach [see below, under feedback for remote coaching])	“It feels good; we (coach and ASHA) talk like friends; I tell her everything, now the problems seem less (severe) than before.” “She talks to me like we talk to friends. It’s not like the coach is there to teach me, it is like that she is there to listen to our problems.” “I share everything with Ritu ji. Even the things which I cannot tell anyone, I share it with her.” “If I am not able to understand, then the coach talks through giving examples. It is easy to understand through examples.”
Overall experience of remote coaching	Experience	Most ASHAs have commented on what they felt—for example, ASHAs summarised their post-coaching state as a feeling of ‘lightness’, ‘relaxation’ or ‘goodness’, or that the calls function as a ‘mood refresher’ or ‘stress-reducer’, or helping them to ‘function (in routine work) without hesitation (building their confidence)’. Certain ASHAs, although finding the calls helpful, could not quote/recall specific examples of how they felt the calls helped. A few ASHAs emphasized on the support the calls gave in problem-solving or learning new things (concepts/ideas) or information Some ASHAs even told that they have learned how to send messages using a mobile phone from the experience of remote coaching calls	“A glimmer of hope is there that the coach will call; madam (coach) asks about how things have been, asking with such attention to detail. In today’s times, who converses in such a manner? It refreshes one’s mood.)” “I felt good, the tension got reduced—due to training, my surroundings feel better.” “I have learned, how to send messages, and through these messages the talk with someone can be done—this has been taught by the coach”
	Suggestions	When asked about any suggestion on the remote coaching calls, none of the ASHAs gave any suggestion but a few even suggested that there is no need for improvement in the existing format, or they seemed hesitant to provide suggestion to the intervention team (reflecting power dynamics mentioned under ‘connection with the coach’)	“There is no need for any improvements in the remote coaching.” “How can I say (any suggestion)?”
Retention of content/knowledge	Recalling CS/Strategy names	Most of the ASHAs were not able to recall the names of strategies they used or applied but were able to tell the steps of the strategies. Some ASHAs remembered the names like Prioritization; Three Funny things; Mindfulness; and Three good things. When asked about the CSs they remembered, all ASHAs were able to name a few CS, for example, self-regulation, kindness, social intelligence, bravery, fairness, and critical thinking/judgment. When telling the names of CSs, they also named some virtues as CS, suggesting less clarity between CS and virtues. Some ASHAs could not recall any strategy name or its steps One ASHA said that the content was related to psychology and another ASHA found the concept of CS and its application to be new	“Earlier I did not know the name of these qualities (CS), and did not identify them (CS) that I may have used (in life, in work), but now I can identify these.” “I have learnt them (strategies), but I don’t remember. I am not able to remember anything.” “Yes, honesty is there, bravery is there, we have to explain to them (the service beneficiary). Perspective (character strength) is used in explaining things to beneficiaries.”
Application of acquired knowledge	Applying strategies learnt in coaching	ASHAs have found utility in applying some strategies suggested in coaching. For instance, mindfulness has helped them relax, reduced nervousness, calmed them after a conflict/quarrel, or helped them after getting free from work, focused their attention and helped ‘forget their problems’. Few ASHAs highlighted issues of getting enough physical space to conduct a mindfulness activity (reflecting on the coaching—because a mindfulness activity need not require enough physical space and can be conducted in the flow of daily activities). Emotional regulation strategies have helped them to keep their cool with seniors, control emotions and modify behaviours in heated conversations. Prioritisation strategies have helped them ‘feel good’, distribute tasks, improve work efficiency, and change certain behaviours (e.g., waking up earlier in the morning). In some cases, ASHAs have provided more general feedback on strategy application without naming a particular strategy (partly because they couldn’t recall during the interview), for instance, strategies helped them ‘reduce anger’ or ‘improve work efficiency’ or ‘manage challenges’. Finally, some ASHAs pointed to other strategies being used such as ‘three funny things’ where an ASHA used it to recall a certain instance with a relative, or an instance involving mimicry	“Sir (health system official) used to say about the work (a number of tasks), but now after making the list, I remember everything. Small tasks are there that I give to my kids to complete. Now I am able to complete my work in a day, by waking early and making a list.” “Breathing exercise (mindfulness) I used to do on the terrace (when the coaching calls were going on) when alone, it reduces nervousness.”
	Applying character-strengths	ASHAs shared instances of use of particular strengths, such as team-work, fairness, social intelligence, self-regulation, bravery and forgiveness, without necessarily using the VIA terms used to denote strengths. A few ASHAs also narrated the use of strengths but without correctly naming them/not recalling the strength’s name during the telephonic interview. The strengths with corresponding examples of application are described as follows: a) team-work/fairness (“taking vote of women participants of the community when planning a meeting (e.g., for any health awareness topic)”; b) social intelligence (e.g., “talk after thinking and understanding things”; “in case of domestic violence, talking to the husband to discuss the matter in front of everyone”); c) self-regulation (e.g., managing her anger; “staying silent in a fight-like situation”); d) forgiveness (e.g., forgetting someone’s mistake) and e) bravery (e.g., supporting a patient to not pay extra out-of-pocket payments)	“Yes, I used to get angry before—I have learned it from the coaching that anger should be reduced and I (tend to) forget if someone makes a mistake.” “I was able to help a child with a task, when people around him were scolding (abusing) him—then I made them understand calmly. Once, they (hospital staff) were asking for INR 500 from a patient for making the (registration) card; but I said no (as the card making is free of charge). This training has helped me (to do all this).”

While we have explained the overall feasibility and acceptability of the remote coaching intervention through results of qualitative data analysis in Sect. 4.6, the findings in Tables 10 and 11 speak to important factors of the ASHA’s context. For example, while the overall logistics of the coaching calls seemed favorable to ASHAs (e.g., less instances of rescheduling and preference for at least 30-min calls with the coach despite their busy schedule), privacy to discuss work-stress issues was a challenge. ASHAs had to ensure that they were in a quiet/silent place when they attended their calls. This suggests that work-stress issues may have overlaps with domestic stress (e.g., stress due to balancing work and home responsibilities) and separating the two domains for a work-stress reduction intervention for rural health workers may not be practical or prudent. The approach of discussing the ways to cope with/address work-stress should therefore involve an open discussion of all related issues from an ASHA’s standpoint. Second, most ASHAs were unable to remember the specific names of character strengths (CS)-based strategies, or the CSs, and some confused between CSs and underlying virtues. However, in terms of utility and application, ASHAs explained how strategies such as mindfulness, emotional regulation and prioritization helped them address work-stress situations, and the steps involved in using certain strategies (e.g., prioritization). This shows that content retention, while being relevant for technical trainings of ASHAs such as administration of a vaccine, may not be the most significant measure of a stress-reduction intervention’s perceived utility. Utility and applicability can be assessed by probing ASHAs on instances where they used a particular strategy in a routine situation, and how it benefitted them, as done through the qualitative interviews of this study. In our fully-powered trial, we plan to conduct two rounds of focus groups—round-1 to explore experiences of workshop and remote coaching for ASHAs who have completed 3-month follow-up, and round-2 to explore sustained application of strategies learnt for ASHAs who have completed 6-month follow-up. Third, ASHAs had little/no suggestions to offer for the format/content of the coaching, after probing, and given that the interviewing team was not involved in delivery of remote coaching. This could be influenced by ASHAs seeing perceived value in the applicability of strategies learnt and their comfort with overall call logistics; however, it may reflect longstanding attitudes of ASHAs shaped by their regular trainings conducted by government agencies, which offer little scope for feedback/suggestion. These trainings are didactic, technical, and topic-specific (e.g., providing antenatal care or immunization), and further, ASHAs’ low hierarchical positions contribute to less feedback to the (senior and experienced) trainer. In this intervention, even with the coaches creating a supportive communicative environment (hence, being able to discuss personal work-stress issues), ASHAs could not fully break the ice due to their possible perception that coaches are like ‘expert trainers’, which may have hindered their critical assessment of the intervention.

### Manipulation Check Results

Twenty-five ASHAs reported feedback form data out of 29 who received the workshop and 18 of these (72%) showed improvement in wellbeing (AHI) scores from baseline to follow-up (mean change: + 12.5 points on AHI, SD: 10.89). Mean feedback score (n = 25 ASHAs) was 25.44 (SD: 2.92, range: 18–30, maximum possible score: 33). Thus, on average, ASHAs were satisfied with the workshop content and delivery to the order of 77%. Pearson correlations revealed the following results: a) relationship between feedback score and ‘delta’ or change (in magnitude) in AHI scores from baseline to 3-month follow-up (n = 25 ASHAs) was 0.11(*p* = 0.6031) and b) for the 18 ASHAs who showed *improvement* in AHI scores from baseline to follow-up, the correlation was 0.14 (*p* = 0.5757).

## Discussion

This pilot study evaluated the preliminary effectiveness of a character-strengths based coaching intervention in comparison to routine (health system) supervision on subjective wellbeing of rural ASHAs in India. The study also aimed to inform modifications and refinements to the coaching intervention in preparation for a large-scale fully powered effectiveness trial (2023–2025). While we previously demonstrated the feasibility and acceptability of the residential workshop component of the intervention (Khan et al., 2023), the current study expanded on our prior work by exploring the feasibility and acceptability of the remote telephonic coaching component and the perspectives of ASHAs towards the intervention in their real world community settings.

### Primary Outcome Analysis

To address our research question and given the gap in literature (section-1), the results of the between-arm difference in authentic happiness scores at 3-month follow-up demonstrated the preliminary effectiveness of a structured positive psychological intervention leveraging personal character-strengths to build the abilities of rural ASHAs’ to cope with work stress to improve their wellbeing.

We found that using the unpaired t test, the total scores on the authentic happiness inventory at 3-month follow-up were significantly higher among ASHAs receiving the intervention relative to the control arm. This is a promising finding, suggesting that character-strengths based coaching in addition to routine supervision is more effective than routine supervision alone in improving subjective happiness levels among ASHAs. AHI findings are also reportedly less susceptible to ceiling effects and more sensitive to upward changes in happiness brought about by a positive psychological intervention (Proyer et al., 2017). As seen earlier, the improvement in AHI items indicates associations with other aspects of routine ‘wellbeing,’ such as the ASHA’s orientations to happiness and positive emotions such as hope, interest, joy, or pride, and further, the items of AHI cover all aspects of hedonic (pleasure-seeking) and eudaimonic (meaning and purpose seeking) wellbeing (Proyer et al., 2017). The finding also indicates the role played by the individual components of the intervention in affecting ASHA’s wellbeing, including the residential workshop, which offered a respite for ASHAs from their routine work to discuss ways of addressing work stress by the use of personal strengths-based coping approaches (Khan et al., 2023), and the continued remote coaching support through weekly telephone calls for nearly two months, which helped reinforce the workshop learnings and provide a safe space for ASHAs to discuss their routine work-stress issues with the coach they had earlier met/interacted with during the workshop. Further, the results also indicate that routine supervision of ASHAs usually focuses on ‘mentoring’ them to improve their effectiveness, through strategies such as experience-sharing and peer-learning (National Health Mission, 2005–2012; Panwar et al., 2012), and these strategies may not necessarily involve an individual-level work stress-reduction component. It is notable that the scores on AHI were obtained with relatively modest sample sizes (intervention arm = 30; control arm = 31), with an effect size (Cohen’s d) of 55%. In the absence of other contextual wellbeing studies for similar rural frontline health workers, we compared these results with studies used for power calculations for our fully-powered trial, and found lesser observed effect size; for example, a 12-week positive psychology intervention with German/Swiss adults (more than two thirds being women) achieved improvements in scores on the authentic happiness inventory at post-test (12 weeks from baseline, where the intervention involved 5 group training sessions on engagement, meaning-making, meditation and philosophical roots of pleasure-seeking) but with an effect size (between-group difference) 10% lesser than the present study (Proyer et al., 2016). Finally, the between-arm difference in AHI scores at 3-month follow-up was observed in presence of comparable social and demographic characteristics of ASHAs at baseline, and given that the pilot study was conducted with ASHAs within the same district over a fixed time period (November 2022 to June 2023), the global health system effects (e.g., delays in monetary incentives, or any general strike leading to stoppage of ASHAs’ delivery of services) would have applied to all ASHAs enrolled in both arms of the study.

However, we found an interesting pattern of AHI scores at 6-month follow-up, approximately three months after the completion of the intervention activities (see Table 4): the mean total AHI score among the intervention arm ASHAs almost remained the same at 6-month follow-up (83.03, SD: 13.23), relative to the 3-month follow-up (83.6, SD:13.32), which shows that wellbeing levels remained stable in the intervention arm at longer-term follow-up, after the stoppage of remote coaching calls. This has been supported by earlier evidence on happiness levels improved by a positive psychological intervention that remained relatively stable over time (Kaczmarek et al., 2015). Besides the statistical non-significance between-arms at 6-month follow-up, we did not gather additional qualitative data from ASHAs of the control arm (as we did in the intervention arm), to obtain insights on their experiences during the study period, which could have plausibly explained the change of wellbeing levels among control arm ASHAs between baseline and 3-month (decline in scores) and 3- and 6-month follow-up (increase in scores, but to attain levels lower than baseline).

### Extrinsic Outcomes Analysis (Burnout and Motivation)

With respect to burnout (emotional exhaustion) and motivation, we have observed no significant differences between arms at follow-up. The means of the total emotional exhaustion scores were all in the 19–26 range (‘moderate burnout’, Maslach & Jackson, 1986) at baseline, 3-month and 6-month follow-ups (group means) for both arms. While these group means were lower in the intervention arm at 3-month follow-up, they did not significantly differ from the control arm (Table 4). This could be partly explained by smaller sample sizes, but also because emotional exhaustion in ASHAs, relative to authentic happiness/subjective wellbeing, could be more closely related to difficult work-structures and workloads of ASHAs’ jobs, which we observed in our formative focus group discussions with ASHAs published elsewhere (Shrivastava et al., 2023), and the strengths-based coaching intervention consisting of a 5-day residential workshop and 8- to 10-week remote telephonic coaching may have fallen short to significantly reduce the adverse effects of the ASHA’s inherent and long-standing work-structure/workload on her emotional wellbeing. This is also reflected in the items of the emotional exhaustion sub-scale, such as “being emotionally drained,” “used up,” “fatigued in the morning,” or “working too hard.” (Maslach & Jackson, 2012). However, it should be mentioned that the two intervention coaches during the pilot (who conducted remote coaching) were newly recruited and had received only a classroom training prior to the delivery of remote coaching. The fully powered trial will involve a team of eight coaches, who will receive classroom training, and an ‘internship’ where they will remotely coach ASHAs of a non-study area, to strengthen their coaching skills before delivering the coaching in the main trial. We believe that with improved coaching skills and improved absorption of coaching content (i.e., use of strengths-based strategies by ASHAs in their routine work) and a larger sample size in the trial, we could potentially see further reductions in the emotional exhaustion scores in the intervention arm (compared to the pilot), and consequently, a significant between-arm difference at 3-month follow-up.

With respect to the motivation measure, we observed substantially higher baseline motivation scores overall (~ 72, Table 4), than the scores reported in other community health worker studies in India (median (IQR) motivation score of 61 (58–64) out of a maximum score of 92) (Tripathy et al., 2016)). These scores may reflect a socially desirable response pattern of community health workers to the items of the instrument (e.g., self-rating their agreement to items such as “I feel motivated to work hard” or “Overall, I feel very satisfied with my job”). The social desirability could be in part due to the perceived influence of the research team on the assessment (despite the instructions and team training in conducting the assessments, and clarification of queries posed by ASHAs, and the emphasis given on ‘no right or wrong answer’), or a positive self-concept (Judge et al., 1998), given the performance monitoring aspect ingrained into an ASHA’s working life. Further, the motivation scores did not show a between-arm significant difference at 3-month follow-up; although, overall, the scores remained more stable in the intervention arm from baseline to 6-month follow-up, relative to the control arm (Table 4). We interpret these results with caution given the small sample sizes.

### Role of Affect, Self-Efficacy, and Flourishing

We aimed to secondarily (descriptively) explore the role of affect, self-efficacy, and flourishing in the relationship between receiving a strengths-based coaching intervention and subjective wellbeing at 3-month follow-up. Overall, in both arms, the positive affect scores marginally improved (i.e., increase in scores) from baseline to 3-month follow-up. Comparing baseline and 3-month follow-up, the negative affect scores marginally increased in the control arm and decreased in the intervention arm. The between-arm differences at 1-month and 3-month follow-up for both positive and negative affect were non-significant. Given the significantly higher wellbeing scores in the intervention arm at 3-month follow-up, and the changes in wellbeing scores between baseline and 3-months (increases in the intervention arm and decreases in the control arm), the above results show a poor correlation between an ASHA’s affect and her subjective wellbeing, potentially indicating the role of multiple factors/stressors—other than the intervention—that could influence the change in affect.

In terms of ASHA’s perceived self-efficacy, the scores showed a (non-significant) increase in the control arm from baseline to 3-month follow-up but not in the intervention arm. The observed lower scores in the intervention arm at 3-month follow-up (with a non-significant between-arm difference) could potentially be explained by an exposure to ‘new’ strengths-based strategies and their usage, aimed at coaching an ASHA to negotiate with work-stress, with a follow-up on the usage of these strategies during successive weekly calls with the coach. The study did not gather additional qualitative data from the intervention arm ASHAs to specifically probe this aspect, and therefore, it is difficult to support the claim that the ‘new knowledge’ of character-strengths (other than the regular [familiar] service delivery trainings that ASHAs usually receive) and its application to reduce work-stress may have had a diminishing effect on self-efficacy.

‘Flourishing’ extends beyond the concept of subjective well-being—or ‘feeling good or functioning well, and/or doing good for others’ (Chaves, 2021)—and reflects a state in which all aspects of an individual’s life are good (Weziak et al., 2019). The several domains of flourishing include happiness and life satisfaction; physical and mental health; meaning and purpose; character and virtue; and close social relationships (included in the ‘Flourish Index (FI)’ scores); and moreover, financial and material stability, while not an end in itself, may have an important role in the sustenance of flourishing (included in the ‘Secure Flourish Index (SFI)’ scores). The study offered an opportunity to gather data on FI and SFI scores in the light of the observed changes in wellbeing (AHI scores) and the patterns were expectedly dissimilar. Both the FI and SFI scores showed non-significant between-arm differences at 1-month and 3-month follow-up. Therefore, the intervention, while playing a role in affecting (current) subjective wellbeing and functioning, did not affect an ASHA’s perception of her flourishing.

### Health-Related Quality of Life

The EQ-5D-5L index scores and visual analogue scale scores were calculated using the norms for Indian population (Jyani et al., 2023), and the group means and effect sizes computed for each time-point (Table 4) showed non-significant between arm differences at 3-month and 6-month follow-up. These results can be justified on the same lines as the results of the FI and SFI, owing to the contribution of systemic factors, particularly the ASHA’s unstructured work, workloads, strained relationships with other cadres, and domestic pressures (Shrivastava et al., 2023), on her self-reported ‘mobility,’ ‘self-care,’ ‘pain and discomfort,’ ‘usual activity’ and ‘anxiety/depression,’ thereby affecting her index scores.

### Self-Perceived Character Strengths (Exploratory Outcome)

Results of paired *t* tests to explore any pre-intervention (before the workshop) and post-intervention (after the last coaching call) differences in self-perceived character-strengths showed a 12.8 point statistically significant difference, indicating enhanced overall perception of strengths in the intervention arm. Significant improvements in these scores noted for certain individual strengths such as forgiveness, spirituality, social responsibility, and open-mindedness, also concur with the personal attributes/strengths reported by ASHAs during our formative focus groups, which serve as sources of motivation to work despite the various stressors (Shrivastava et al., 2023), and these could be a combination of ‘realized’ and ‘unrealized’ strengths developed during the course of many years of community work (Mackie, 2014).

### Feasibility and Acceptability of the Remote Coaching Intervention

Out of the ASHAs targeted to receive the workshop, 83% attended it (inducted into the intervention) and of these, 83% received the optimum ‘dose’ of the remote coaching calls (or, at least 8 calls). While these figures show engagement, the post-pilot telephonic interviews with ASHAs revealed important findings: First, ASHAs demonstrated an interest in receiving the remote coaching support calls, in part due to the priority given by the coach (as part of study protocol) to the ASHA’s available day and time, which was reflected in less instances of call rescheduling, preferences of ASHAs for discussing their issues with the coach in their private (domestic) space, wanting the call to last for a substantial length of time (being comfortable with calls up to 45 min or an hour, with calls typically lasting for at least 30 min), wanting to receive calls every 7–10 days, and also indicating a desire to receive more than eight calls (or longer-term continued coaching support). Second, while exploring ASHAs’ perceptions on call reminders, we noted an inclination towards telephone reminders than reading the messages sent by the coaching team on WhatsApp, due to challenges in reading text and smartphone literacy. This informs future intervention approaches to rely more on simpler means of interfacing with ASHAs, given the rural context and it suggests that despite the use of smartphones and chat messages in their working spaces, ASHAs and similar rural health workers may still prefer to interact over telephone calls to discuss work-stress issues. A previous contextual study also relied on telephonic support to address challenges of ASHAs in receiving digital training on the delivery of community-based brief psychological treatment for persons with depression (Naslund et al., 2021). Third, ASHAs have expressed varying forms of ‘connection’ with the coach; for example, the coach is like their ‘friend or sister,’ someone who ‘unburdens’ them, someone who is a mentor/confidant, or has good skills in explaining things to them. These connections have been felt in spite of the unavoidable effects of power dynamics between an ‘external’ coach perceived as a ‘senior’, partly due to the fact that ASHAs are accustomed to supervision, and work at the bottom of hierarchies. These connections could be influenced by gender—both the pilot coaches were women—but the practices emphasized in coach training also have a crucial role to play i.e., the emphasis on warmth/empathy, active listening, collaborative discussion, summarizing the ASHA’s narrative and showing genuine praise for the ASHA’s efforts in negotiating with stress/using the coaching strategies. These soft skills have been an integral part of assessing therapist competence in the Indian context (Kohrt et al., 2015), and influenced the coach supervision checklist, which was developed by the team and used in the pilot (Sect. 2.7.2). The felt connections were formed, in part by the contrasting relationships ASHAs develop with their regular trainers (senior cadres of the health system) during the course of their (more didactic, instructional, and hierarchical) service delivery trainings. Fourth, ASHAs found utility in some of the coaching strategies that they discussed during the remote calls, which was reassuring from the perspective of a novice form of strengths-based coaching support piloted in this study; for example, *emotional regulation strategies* helped them to ‘keep their cool with seniors, control emotions and modify behaviours in heated conversations’, *prioritisation strategies* helped them ‘feel good, distribute tasks, improve work efficiency, and change certain behaviours (e.g., waking up earlier in the morning)’, and *mindfulness-based strategies* helped them to ‘reduce nervousness, keep calm and focus their attention’. In line with the aforesaid practices highlighted in coach training, we attribute these findings to the coach’s efforts of active listening, selecting and matching a strategy with the type of ASHA’s stress-narrative, ‘breaking’ down strategies into simpler steps, and following up on their usage in successive calls, emphasizing on ASHA’s perceived abilities, and ways to achieving her goals despite obstacles. These coaching approaches build on the core concepts elucidated in Snyder’s cognitive model of hope (Snyder, 2002) i.e., goals, pathways, and agency, where *goals* are mental targets that guide human behaviors (e.g., as an ASHA, I have to complete multiple tasks related to vaccination and prenatal check-ups, plus domestic duties and preparing for guests coming at home, over next ten days), *pathways* involve perceived abilities to generate multiple routes to desired goals (e.g., I can prepare a list, decide what’s important but also what’s important and urgent, serially order my tasks, and delegate some tasks to family members at home/neighbors if possible), and *agency* involves perceived abilities to initiate and sustain movement along a pathway (e.g., I can stick to my ‘list’ and follow along, as ‘social responsibility’ is my [character] strength, and I don’t have to feel that this is something I cannot do or keep up with).

### Limitations

This study has several limitations. *First,* in the results of the manipulation check, while the ASHAs showed a 77% degree of satisfaction with the workshop content and delivery, the correlation between feedback for the workshop and change in wellbeing scores from baseline to 3-month follow-up was small and not significant. While this may be due to small sample sizes, we have to account for other factors that could have influenced wellbeing scores, apart from ASHA’s overall perception of the workshop; for example, the application of workshop learnings in routine life, which would have required more direct observations or detailed experience gathering, which was not possible given the study design. Doing these observations may have also incurred a social desirability bias for the ASHAs’ responses, given the hierarchically low work-positions of ASHAs. Therefore, apart from the manipulation check, we also conducted qualitative telephonic interviews to understand the experience of the intervention and analyzed this data using thematic analysis, as discussed in Sect. 2.8.2.1*. Second,* improvements in AHI scores may correlate to other meaningful aspects of routine wellbeing of ASHAs such as orientations to happiness and experience of positive emotions (e.g., hope, interest, joy, or pride), but the study did not specifically assess these measures and therefore, any possible changes in these outcomes could not be attributed to the intervention. Future studies should address this gap and link the improvements in the AHI with work function and a health worker's overall engagement and retention in the workplace. *Third,* improvements in AHI scores reflected changes in ‘current’ happiness levels of ASHAs, as a response to the intervention, but the intervention was not designed to address the larger structural issues contributing to ASHAs’ work stress such as excessive workloads, unstructured work, unmet capacity-building needs, low and untimely incentives, role confusion and effects of poverty, and patriarchy (especially at home). This is partly reflected in the results of flourishing scores, quality of life indices, and burnout scores, which were largely not impacted by the intervention. We recommend the health system to take urgent measures to address these structural issues, such as the recent step to upgrade the ASHA payment grades (Hindustan Times, 2023) by the state government of Madhya Pradesh, where the study was conducted. However, given the nature of ASHAs’ work, multiple responsibilities, multiple engagements with different personnel and community members, and the recurrent emotional labor, the levels of her current happiness can be moderated by a wellbeing-enhancing intervention, and in this respect, it is crucial to see that the scores on AHI improved in the intervention arm at 3-month follow-up and maintained at 6-month follow-up. Based on the results of the fully powered trial (2023–2025), if these results are replicated with substantial effect sizes, we will propose the government to continue character-strengths coaching support over a longer time period, or preferably, on a continuous basis, as ASHAs have suggested in the qualitative interviews (Table 10), through health system appointed intervention coaches. This will address their current happiness, as structural measures continue to be put in place to address the larger problems of their workspace to improve their motivation and retention (Singh et al., 2015; Willis-Shattuck et al., 2008). In this respect, we want to highlight the (published) findings of our formative research (Shrivastava et al., 2023), which discuss how the ASHAs see themselves and the complex self-evaluation of their work, and how do the social and structural factors embedded in their work, role of gender/patriarchy, and socio-economic strain affects their stress – for example, recognizing/valuing that their work contributes to reducing mortality (e.g., maternal mortality), even with the attendant emotional labour in instances of death or disease which contribute to acute stress and rumination for the ASHA (who’s handling the case), or, family members ridiculing their long work-hours or putting pressure on ASHAs to discontinue working due to less pay and giving less time at home (for their children/husbands), while the cadre receives respect from the village-community for their efforts. While this pilot study and the forthcoming trial emphasize on improving levels of wellbeing/current happiness, we recognize that these larger structural issues cannot be separated, and our efforts at disseminating the trial results will take necessary steps to build the narrative that the health system is primarily tasked with reducing the structural stressors of ASHAs, while strengths-based interventions help improve their coping with routine stress. *Fourth,* while the sample size proved adequate to test the between-arm difference in wellbeing at follow-up, and the sample was drawn randomly from the district population of ASHAs, the ASHAs who engaged with the intervention and particularly those who received the full dose of eight remote coaching calls, may have been interested (intrinsically) in receiving such coaching. Therefore, they may not represent the groups of ASHAs living with severe work stress, who may refrain from putting substantial time and effort in attending a 5-day residential workshop and discussing their problems with an external coach for over two months of weekly calls. *Fifth,* the team incurred human resource challenges in coding the post-pilot qualitative interview data and one dedicated coder (SA) was assigned for the same, supervised by a study co-investigator (APB) with expertise in qualitative analysis. A greater number of primary coders could have minimized the biases introduced by the single coder through the stages of thematic analysis, such as familiarizing with the interview notes, forming initial categories of information, developing codes, and cross-analysis of codes to generate themes. *Sixth,* we tried to minimize social desirability biases in the responses of ASHAs—as also observed in prior contextual studies (Muke et al., 2020)—towards the questions of these post-pilot interviews by excluding the facilitators of the intervention workshops (AK, LS, other members of the intervention development team) in the interview process; however, the aforementioned coder (SA) had played a supportive role in the delivery of these workshops, and as such, the ASHAs were familiar with her, which could have made their answers socially desirable. To reduce social desirability in the main trial, we will involve a separate team of moderators who will conduct detailed focus group discussions to gather experiences of ASHAs of both the workshop and remote coaching. *Seventh,* we found significant improvements in the self-perception of certain character-strengths before and after receiving the intervention (8 out of 24 strengths) and a significant change in the total self-perceived strengths score, but without a similar assessment in the control arm, we cannot demonstrate that these changes are contributed by the intervention i.e., addressing work-stress through strengths-based coaching and the improved wellbeing levels at 3-month follow-up are associated with an increased self-perception of strengths. We plan to conduct these strengths assessments pre- and post in both arms of the main trial. *Eighth,* we did not stratify the randomization owing to an (anticipated) balance of social and demographic factors such as ASHA’s age and education levels at baseline (Table 2), but due to a possible moderation of the absorption of intervention content, and thus the wellbeing outcome, by ASHA’s age and education level, and a likely enhancement of the power of the design of trials with < 400 participants (Kernan et al., 1999), we plan to stratify the randomization in the main trial by age and education levels of ASHAs. *Finally,* ASHAs, who work at the individual village-level, were recruited from real-world rural settings where it is not possible to fully reduce contamination (Keogh et al., 2007) as ASHAs may interact with each other in frequent encounters in their workplace i.e., meetings at primary health centres, or socially, by residing in adjacent villages.

## Ethical Considerations

All study procedures have been approved by Sangath Institutional Review Board (Ethical review number: AB_2021_72). ASHAs who were invited to participate in the study through the study introduction and baseline assessment completed a written informed consent form. All participants’ records of assessments and qualitative interviews were be kept in password-protected computers. All physical copies of documents were kept in locked cabinets located inside the Sangath office in Bhopal. All data containing personal identifiers of participants were delinked—we fully anonymized personal data by removing all direct identifiers and assigned a unique identification number combining all main indirect identifiers. From the perspective of anticipated risks, we understood that ASHAs may find talking about or reporting their burnout or work-stress symptoms difficult and distressing, during outcome assessments and/or interactions with intervention coaches. Hence, outcome assessors and intervention coaches were specifically trained on how to address emotional distress in such situations, including if needed, referral to professional help, for example, the psychiatrist at the district hospital via members of the Bhopal study team (AK, AS, SS) who are trained psychologists. Processes were put in place such that the study team members as needed could individually speak with the ASHA and discuss her concerns before formal professional support at the district hospital was organized.

## Conclusions

The findings from this pilot study offer insights related to preliminary effectiveness, feasibility and acceptability of a character-strengths based coaching intervention to improve wellbeing and reduce work stress among rural community health workers in India, which are relevant in preparation for a larger fully powered effectiveness trial. An important finding from this pilot study was the significant improvement in health workers’ happiness levels at follow-up, also supported by qualitative inputs by ASHAs on their felt connections with the coaches, usability of coaching strategies in addressing work-stress situations, and perceived ease in coaching call logistics within their real-world settings i.e., attending and putting sufficient time for 8–10 weekly telephonic calls. At the same time, findings of secondary outcome analysis indicate that the intervention had marginal effects on improvement of flourishing and perceived quality of life, which potentially relate to the larger work-structure and workload issues of ASHAs, in addition to the difficulties they experience in their relationships with other cadres, and the village community, and managing their domestic duties. While these larger extrinsic issues need structural solutions and continued investments by the health system into the ASHA program at large, this study was able to demonstrate that the more intrinsic factors such as the individual abilities of ASHAs to manage their emotions in response to outer demands (‘emotional labour’, discussed in section-1), and cope with stressful situations can be systematically ‘coached’ using character-strengths to improve current happiness and subjective wellbeing, and also maintain these improvements over time.

From the standpoint of theoretical implications, this study critically contributes to the broader positive psychology evidence base on the use of character-strengths for building capacities to cope with work-stress among rural frontline workers in low-resource settings. This is especially relevant as longitudinal studies in India are scarce in this space. With respect to practical implications, this study informs our fully powered randomized controlled trial in terms of revising the intervention structure and content (e.g., based on qualitative findings on perspectives of ASHAs) before delivering the intervention to the targeted 270 ASHAs. Results of the trial will be discussed in a dissemination meeting in 2025 with the state health department to discuss the scope of potential adoption of the intervention by the health system for rural ASHAs in Madhya Pradesh.

To conclude, this study and the subsequent trial will critically contribute to the broader literature on character-strengths and inform the ways by which capacities of rural frontline workers to use character strengths to improve wellbeing can be developed in low-resource settings.

## Supplementary Information

Below is the link to the electronic supplementary material.

Supplementary file1 (DOCX 79 KB)

Supplementary file1 (DOCX 24 KB)

## Data Availability

Raw data for study quantitative and qualitative datasets are not publicly available to preserve individuals’ privacy under the General Data Protection Regulation.
