# Supplementary material for: Evaluation of a Positive Psychological Intervention to Reduce Work Stress among Rural Community Health Workers in India: Results from a Randomized Pilot Study
Source: J Happiness Stud. 2025 Feb 17;26(3):27. doi: 10.1007/s10902-024-00852-6 (PMC11832669; doi:10.1007/s10902-024-00852-6)
Supplement: Supplementary file 1 — Supplementary file1 (DOCX 79 KB) [file 10902_2024_852_MOESM1_ESM.docx]

Supplemental tables

Expanded results of ANCOVA analysis and repeated measures ANCOVA

Table 1- AHI

| ANCOVA at 3 month follow-up: | |  |  |  |  |
| --- | --- | --- | --- | --- | --- |
| **Source of variation** | **SUM OF SQUARE** | **DF** | **MEAN SUM OF SQUARE** | **F-statistics** | **p-value** |
|  |  |  |  |  |  |
| Model | 1629.1427 | 2 | 814.57134 | 4.96 | 0.0103 |
| Arm* | 961.36013 | 1 | 961.36013 | 5.86 | 0.0187 |
| AHI score at baseline* | 821.70704 | 1 | 821.70704 | 5.01 | 0.0291 |
| Residual | 9522.2672 | 58 | 164.17702 |  |  |
| Total | 11151.41 | 60 | 185.85683 |  |  |

| ANCOVA at 6 month follow-up: | |  |  |  |  |
| --- | --- | --- | --- | --- | --- |
| **Source of variation** | **SUM OF SQUARE** | **DF** | **MEAN SUM OF SQUARE** | **F-statistics** | **p-value** |
|  |  |  |  |  |  |
| Model | 3226.5111 | 2 | 1613.2556 | 9.52 | 0.0003 |
| Arm | 179.10234 | 1 | 179.10234 | 1.06 | 0.3083 |
| AHI score at baseline* | 3146.1495 | 1 | 3146.1495 | 18.57 | 0.0001 |
| Residual | 9489.116 | 56 | 169.4485 |  |  |
| Total | 12715.627 | 58 | 219.23495 |  |  |

Repeated Measures ANCOVA:

| **Source of Variation** | **Coefficient** | **SE** | **z-statistic** | | **p-value** | **95% CI** | |
| --- | --- | --- | --- | --- | --- | --- | --- |
| Between Individuals |  |  | |  |  | LL | UL |
| Time | 1.1 | 8.2 | |  |  | 0.0 | 2596403 |
| UID (ASHA) | 54.6 | 53.7 | |  |  | 7.9 | 375.1 |
| Residual | 101.5 | 19.1 | |  |  | 70.2 | 146.7 |
| Within Individuals |  |  | |  |  |  |  |
| Time | 1.9 | 1.9 | | 1.0 | 0.3 | -1.7 | 5.6 |
| Arm | 5.7 | 2.7 | | 2.1 | 0.0 | 0.4 | 11.1 |
| AHI total at baseline | 0.4 | 0.1 | | 4.0 | 0.0 | 0.2 | 0.6 |
| Intercept | 40.5 | 9.7 | | 4.2 | 0.0 | 21.5 | 59.6 |

Table 2- Emotional Exhaustion Subscale of Maslach Burnout Inventory (MBI)

| ANCOVA at 3 month follow up: | |  |  |  |  |
| --- | --- | --- | --- | --- | --- |
| Source of variation | **SUM OF SQUARE** | **DF** | **MEAN SUM OF SQUARE** | **F-statistics** | **p-value** |
|  |  |  |  |  |  |
| Model | 1037.1756 | 2 | 518.58782 | 5.28 | 0.0078 |
| Arm | 36.22177 | 1 | 36.22177 | 0.37 | 0.5459 |
| MBI Score at Baseline* | 983.7457 | 1 | 983.7457 | 10.03 | 0.0025 |
| Residual | 5691.4145 | 58 | 98.127836 |  |  |
| Total | 6728.5902 | 60 | 112.14317 |  |  |

| ANCOVA at 6 month follow up: | |  |  |  |  |
| --- | --- | --- | --- | --- | --- |
| Source of variation | **SUM OF SQUARE** | **DF** | **MEAN SUM OF SQUARE** | **F-statistics** | **p-value** |
|  |  |  |  |  |  |
| Model | 569.66542 | 2 | 284.83271 | 3.48 | 0.0378 |
| Arm | 16.137444 | 1 | 16.137444 | 0.2 | 0.6589 |
| MBI score at baseline* | 555.42374 | 1 | 555.42374 | 6.78 | 0.0118 |
| Residual | 4589.3176 | 56 | 81.952101 |  |  |
| Total | 5158.9831 | 58 | 88.947984 |  |  |

Repeated Measures ANCOVA:

| Source of Variation | Coefficient | Standard Error | z-statistic | p-value | 95% CI | |
| --- | --- | --- | --- | --- | --- | --- |
| Between Individuals |  |  |  |  | LL | UL |
| Time | 0.00 | 0.00 |  |  | 0.0 | . |
| UID | 45.9 | 12.6 |  |  | 26.8 | 78.7 |
| Residual | 40.4 | 7.5 |  |  | 28.1 | 58.0 |
| Within Individuals |  |  |  |  |  |  |
| Time | -0.8 | 1.2 | -0.7 | 0.5 | -3.1 | 1.5 |
| Arm | -0.4 | 2.1 | -0.2 | 0.9 | -4.4 | 3.7 |
| MBI total at baseline | 0.4 | 0.1 | 3.4 | 0.0 | 0.2 | 0.6 |
| Intercept | 15.6 | 4.4 | 3.5 | 0.0 | 6.9 | 24.3 |

Table 3- Motivation Scale

| ANCOVA at 3 month follow up: | |  |  |  |  |
| --- | --- | --- | --- | --- | --- |
| Source of variation | **SUM OF SQUARE** | **DF** | **MEAN SUM OF SQUARE** | **F-statistics** | **p-value** |
|  |  |  |  |  |  |
| Model | 492.76804 | 2 | 246.38402 | 6.94 | 0.002 |
| Arm | 57.991297 | 1 | 57.991297 | 1.63 | 0.2062 |
| MOTIVATION score at baseline* | 449.09669 | 1 | 449.09669 | 12.66 | 0.0008 |
| Residual | 2057.7893 | 58 | 35.479126 |  |  |
| Total | 2550.5574 | 60 | 42.50929 |  |  |

| ANCOVA at 6 month follow up: | |  |  |  |  |
| --- | --- | --- | --- | --- | --- |
| Source of variation | **SUM OF SQUARE** | **DF** | **MEAN SUM OF SQUARE** | **F-statistics** | **p-value** |
|  |  |  |  |  |  |
| Model | 389.73266 | 2 | 194.86633 | 4.97 | 0.0103 |
| Arm | 23.198356 | 1 | 23.198356 | 0.59 | 0.4449 |
| MOTIVATION score at baseline* | 354.69894 | 1 | 354.69894 | 9.05 | 0.0039 |
| Residual | 2194.9114 | 56 | 39.194847 |  |  |
| Total | 2584.6441 | 58 | 44.562829 |  |  |

Repeated Measures ANCOVA:

| Source of Variation | Coefficient | Standard Error | z-statistic | p-value | 95% CI | |
| --- | --- | --- | --- | --- | --- | --- |
| Between Individuals |  |  |  |  | **LL** | **UL** |
| TIME | 0.8 | 1.8 |  |  | 0.01 | 85.89 |
| UID | 5.4 | 11.9 |  |  | 0.07 | 409.11 |
| Residual | 25.8 | 4.9 |  |  | 17.80 | 37.30 |
| Within Individuals |  |  |  |  |  |  |
| Time | -2.1 | 0.9 | -2.2 | 0.0 | -3.9 | -0.3 |
| Arm | -0.5 | 1.2 | -0.4 | 0.7 | -2.9 | 1.9 |
| Motivation total at baseline | 0.4 | 0.1 | 4.3 | 0.0 | 0.2 | 0.5 |
| Intercept | 50.6 | 6.8 | 7.5 | 0.0 | 37.4 | 63.9 |

Table 4 – PANAS Positive Affect

| ANCOVA at 1 month follow up: |  |  |  |  |  |
| --- | --- | --- | --- | --- | --- |
| **Source of variation** | **SUM OF SQUARE** | **DF** | **MEAN SUM OF SQUARE** | **F-statistics** | **p-value** |
|  |  |  |  |  |  |
| Model | 134.7 | 2 | 67.3 | 2.53 | 0.088 |
| Arm | 3.7 | 1 | 3.7 | 0.14 | 0.709 |
| PANAS positive score at baseline* | 119.5 | 1 | 119.5 | 4.49 | 0.038 |
| Residual | 1568.3 | 59 | 26.6 |  |  |
| Total | 1702.9 | 61 | 27.9 |  |  |

| ANCOVA at 3 month follow up: |  |  |  |  |  |
| --- | --- | --- | --- | --- | --- |
| **Source of variation** | **SUM OF SQUARE** | **DF** | **MEAN SUM OF SQUARE** | **F-statistics** | **p-value** |
|  |  |  |  |  |  |
| Model | 126.8 | 2 | 63.4 | 2.75 | 0.073 |
| Arm | 5.0 | 1 | 5.0 | 0.22 | 0.645 |
| PANAS positive score at baseline* | 112.6 | 1 | 112.6 | 4.88 | 0.031 |
| Residual | 1338.0 | 58 | 23.1 |  |  |
| Total | 1464.8 | 60 | 24.4 |  |  |

Repeated measures ANCOVA

| Source of Variation | Coefficient | Standard Error | z-statistic | p-value | 95% CI | |
| --- | --- | --- | --- | --- | --- | --- |
| Between Individuals |  |  |  |  | **LL** | **UL** |
| Time | 0.00 | 0.00 |  |  | 0 | . |
| UID | 7.5 | 3.3 |  |  | 3.1 | 17.9 |
| Residual | 16.3 | 3.0 |  |  | 11.3 | 23.4 |
| Within Individuals |  |  |  |  |  |  |
| Time | 0.9 | 0.7 | 1.2 | 0.2 | -0.5 | 2.3 |
| Arm | 0.6 | 1.0 | 0.6 | 0.5 | -1.4 | 2.6 |
| PANAS+ Score at baseline | 0.3 | 0.1 | 2.7 | 0.0 | 0.1 | 0.5 |
| Intercept | 19.0 | 3.2 | 5.9 | 0.0 | 12.7 | 25.2 |

Table 5 – PANAS Negative Affect

| ANCOVA at 1month follow up: |  |  |  |  |  |
| --- | --- | --- | --- | --- | --- |
| **Source of variation** | **SUM OF SQUARE** | **DF** | **MEAN SUM OF SQUARE** | **F-statistics** | **p-value** |
|  |  |  |  |  |  |
| Model | 281.1 | 2 | 140.6 | 4.4 | 0.017 |
| Arm | 8.8 | 1 | 8.8 | 0.27 | 0.602 |
| PANAS negative score at baseline* | 233.0 | 1 | 233.0 | 7.3 | 0.009 |
| Residual | 1883.3 | 59 | 31.9 |  |  |
| Total | 2164.5 | 61 | 35.5 |  |  |

| ANCOVA at 3month follow up: |  |  |  |  |  |
| --- | --- | --- | --- | --- | --- |
| **Source of variation** | **SUM OF SQUARE** | **DF** | **MEAN SUM OF SQUARE** | **F-statistics** | **p-value** |
|  |  |  |  |  |  |
| Model | 483.7 | 2 | 241.8 | 10.92 | 0.0001 |
| Arm | 44.6 | 1 | 44.6 | 2.01 | 0.1612 |
| PANAS negative score at baseline* | 478.7 | 1 | 478.7 | 21.61 | 0.0000 |
| Residual | 1284.7 | 58 | 22.1 |  |  |
| Total | 1768.3 | 60 | 29.5 |  |  |

Repeated measures ANCOVA:

| Source of Variation | Coefficient | Standard Error | z-statistic | p-value | 95% CI | |
| --- | --- | --- | --- | --- | --- | --- |
| Between Individuals |  |  |  |  | **LL** | **UL** |
| Time | 0.00 | 0.00 |  |  | 0 | . |
| UID | 12.4 | 3.7 |  |  | 6.9 | 22.4 |
| Residual | 13.8 | 2.5 |  |  | 9.7 | 19.8 |
| Within Individuals |  |  |  |  |  |  |
| Time | 0.3 | 0.7 | 0.4 | 0.7 | -1.1 | 1.6 |
| Arm | -0.5 | 1.1 | -0.4 | 0.7 | -2.7 | 1.8 |
| PANAS- Score at baseline | 0.4 | 0.1 | 4.2 | 0.0 | 0.2 | 0.5 |
| Intercept | 16.8 | 2.7 | 6.2 | 0.0 | 11.5 | 22.1 |

Table 6 – Self Efficacy

| ANCOVA at 1 month follow-up: |  |  |  |  |  |
| --- | --- | --- | --- | --- | --- |
| Source of variation | **SUM OF SQUARE** | **DF** | **MEAN SUM OF SQUARE** | **F-statistics** | **p-value** |
|  |  |  |  |  |  |
| Model | 307.0 | 2 | 153.5 | 2.57 | 0.085 |
| Arm | 13.2 | 1 | 13.2 | 0.22 | 0.640 |
| OSES score at Baseline* | 289.3 | 1 | 289.3 | 4.84 | 0.032 |
| Residual | 3523.8 | 59 | 59.7 |  |  |
| Total | 3830.8 | 61 | 62.8 |  |  |

| ANCOVA at 3 month follow-up: |  |  |  |  |  |
| --- | --- | --- | --- | --- | --- |
| **Source of variation** | **SUM OF SQUARE** | **DF** | **MEAN SUM OF SQUARE** | **F-statistics** | **p-value** |
|  |  |  |  |  |  |
| Model | 1318.1 | 2 | 659.1 | 8.51 | 0.0006 |
| Arm | 206.9 | 1 | 206.9 | 2.67 | 0.1075 |
| OSES score at Baseline* | 1109.1 | 1 | 1109.1 | 14.33 | 0.0004 |
| Residual | 4490.1 | 58 | 77.4 |  |  |
| Total | 5808.2 | 60 | 96.8 |  |  |

Repeated measures ANCOVA:

| Source of Variation | Coefficient | Standard Error | z-statistic | p-value | 95% CI | |
| --- | --- | --- | --- | --- | --- | --- |
| Between Individuals |  |  |  |  | **LL** | **UL** |
| Time | 3.4 | 3.6 |  |  | 0.4 | 27.8 |
| UID | 0.0 | 0.0 |  |  | 0.0 | . |
| Residual | 59.0 | 10.9 |  |  | 41.1 | 84.7 |
| Within Individuals |  |  |  |  |  |  |
| Time | -0.5 | 1.4 | -0.3 | 0.7 | -3.2 | 2.3 |
| Arm | -1.2 | 1.5 | -0.8 | 0.4 | -4.2 | 1.8 |
| OSES Score at baseline | 0.3 | 0.1 | 4.1 | 0.0 | 0.2 | 0.5 |
| Intercept | 55.3 | 6.4 | 8.7 | 0.0 | 42.8 | 67.8 |

Table 7 – Flourish Index

| ANCOVA at 1 month follow-up: |  |  |  |  |  |
| --- | --- | --- | --- | --- | --- |
| Source of variation | **SUM OF SQUARE** | **DF** | **MEAN SUM OF SQUARE** | **F-statistics** | **p-value** |
|  |  |  |  |  |  |
| Model | 4.9 | 2 | 2.4 | 1.96 | 0.149 |
| Arm | 1.4 | 1 | 1.4 | 1.12 | 0.295 |
| FI mean score at Baseline | 3.8 | 1 | 3.8 | 3.06 | 0.085 |
| Residual | 73.2 | 59 | 1.2 |  |  |
| Total | 78.0 | 61 | 1.3 |  |  |

| ANCOVA at 3 month follow-up: |  |  |  |  |  |
| --- | --- | --- | --- | --- | --- |
| **Source of variation** | **SUM OF SQUARE** | **DF** | **MEAN SUM OF SQUARE** | **F-statistics** | **p-value** |
|  |  |  |  |  |  |
| Model | 18.5 | 2 | 9.3 | 7.91 | 0.0009 |
| Arm | 1.7 | 1 | 1.7 | 1.42 | 0.2383 |
| FI mean score at Baseline* | 17.5 | 1 | 17.5 | 14.96 | 0.0003 |
| Residual | 67.9 | 58 | 1.2 |  |  |
| Total | 86.4 | 60 | 1.4 |  |  |

Repeated measures ANCOVA:

| Source of Variation | Coefficient | Standard Error | z-statistic | p-value | 95% CI | |
| --- | --- | --- | --- | --- | --- | --- |
| Between Individuals |  |  |  |  | **LL** | **UL** |
| Time | 0.00 | 0.00 |  |  | 0 | . |
| UID | 0.5 | 0.2 |  |  | 0.2 | 0.9 |
| Residual | 0.7 | 0.1 |  |  | 0.5 | 1.0 |
| Within Individuals |  |  |  |  |  |  |
| Time | -0.2 | 0.2 | -1.3 | 0.2 | -0.5 | 0.1 |
| Arm | -0.3 | 0.2 | -1.4 | 0.2 | -0.8 | 0.1 |
| FI mean Score at baseline | 0.3 | 0.1 | 3.4 | 0.0 | 0.1 | 0.5 |
| Intercept | 6.3 | 0.7 | 9.2 | 0.0 | 4.9 | 7.6 |

Table 8 – Secure Flourish Index

| ANCOVA at 1 month follow-up: |  |  |  |  |  |
| --- | --- | --- | --- | --- | --- |
| Source of variation | **SUM OF SQUARE** | **DF** | **MEAN SUM OF SQUARE** | **F-statistics** | **p-value** |
|  |  |  |  |  |  |
| Model | 5.6 | 2 | 2.8 | 2.27 | 0.112 |
| Arm | 0.5 | 1 | 0.5 | 0.43 | 0.515 |
| SFI mean score at Baseline* | 5.4 | 1 | 5.4 | 4.39 | 0.041 |
| Residual | 73.3 | 59 | 1.2 |  |  |
| Total | 78.9 | 61 | 1.3 |  |  |

| ANCOVA at 3 month follow-up: |  |  |  |  |  |
| --- | --- | --- | --- | --- | --- |
| Source of variation | **SUM OF SQUARE** | **DF** | **MEAN SUM OF SQUARE** | **F-statistics** | **p-value** |
|  |  |  |  |  |  |
| Model | 20.6 | 2 | 10.3 | 9.49 | 0.0003 |
| Arm | 1.2 | 1 | 1.2 | 1.07 | 0.3042 |
| SFI mean score at Baseline* | 20.4 | 1 | 20.4 | 18.74 | 0.0001 |
| Residual | 63.0 | 58 | 1.1 |  |  |
| Total | 83.7 | 60 | 1.4 |  |  |

Repeated measures ANCOVA:

| Source of Variation | Coefficient | Standard Error | z-statistic | p-value | 95% CI | |
| --- | --- | --- | --- | --- | --- | --- |
| Between Individuals |  |  |  |  | **LL** | **UL** |
| Time | 0.00 | 0.00 |  |  | 0 | . |
| UID | 0.5 | 0.2 |  |  | 0.3 | 0.9 |
| Residual | 0.6 | 0.1 |  |  | 0.4 | 0.9 |
| Within Individuals |  |  |  |  |  |  |
| Time | -0.1 | 0.1 | -0.8 | 0.4 | -0.4 | 0.2 |
| Arm | -0.2 | 0.2 | -1.0 | 0.3 | -0.7 | 0.2 |
| SFI mean Score at baseline | 0.3 | 0.1 | 3.8 | 0.0 | 0.2 | 0.5 |
| Intercept | 5.7 | 0.7 | 8.6 | 0.0 | 4.4 | 7.0 |

Table 9 – EQ-5D Utility Index

| ANCOVA at 3-month follow up: | |  |  |  |  |
| --- | --- | --- | --- | --- | --- |
| **Source of variation** | **SUM OF SQUARE** | **DF** | **MEAN SUM OF SQUARE** | **F-statistics** | **p-value** |
|  |  |  |  |  |  |
| Model | 0.08889475 | 2 | 0.04444738 | 1.41 | 0.2534 |
| Arm | 0.00235948 | 1 | 0.00235948 | 0.07 | 0.7857 |
| EQ5D Utility score at baseline | 0.08864034 | 1 | 0.08864034 | 2.8 | 0.0995 |
| Residual | 1.8015768 | 57 | 0.03160661 |  |  |
| Total | 1.8904716 | 59 | 0.03204189 |  |  |

| ANCOVA at 6-month follow up: | |  |  |  |  |
| --- | --- | --- | --- | --- | --- |
| **Source of variation** | **SUM OF SQUARE** | **DF** | **MEAN SUM OF SQUARE** | **F-statistics** | **p-value** |
|  |  |  |  |  |  |
| Model | 0.07172865 | 2 | 0.03586433 | 4.51 | 0.0153 |
| Arm | 0.00002114 | 1 | 0.00002114 | 0 | 0.959 |
| EQ5D Utility score at baseline* | 0.06799149 | 1 | 0.06799149 | 8.56 | 0.005 |
| Residual | 0.43700194 | 55 | 0.00794549 |  |  |
| Total | 0.50873059 | 57 | 0.0089251 |  |  |

Repeated measures ANCOVA:

| Source of Variation | Coefficient | Standard Error | z-statistic | p-value | 95% CI | |
| --- | --- | --- | --- | --- | --- | --- |
| Between Individuals |  |  |  |  | **LL** | **UL** |
| TIME | 0.00 | 0.00 |  |  | 0 | . |
| UID | 0.004 | 0.002 |  |  | 0.001 | 0.014 |
| Residual | 0.015 | 0.003 |  |  | 0.011 | 0.022 |
| Within Individuals |  |  |  |  |  |  |
| Time | 0.047 | 0.023 | 2.080 | 0.038 | 0.003 | 0.092 |
| Arm | 0.006 | 0.028 | 0.200 | 0.841 | -0.050 | 0.061 |
| EQ5D Utility Score at baseline | 0.419 | 0.159 | 2.630 | 0.008 | 0.107 | 0.731 |
| Intercept | 0.356 | 0.161 | 2.210 | 0.027 | 0.040 | 0.672 |

Table 10 – Visual Analogue Scale (VAS)

| ANCOVA at 3 month follow up |  |  |  |  |  |
| --- | --- | --- | --- | --- | --- |
| **Source of variation** | **SUM OF SQUARE** | **DF** | **MEAN SUM OF SQUARE** | **F-statistics** | **p-value** |
|  |  |  |  |  |  |
| Model | 1327.2 | 2 | 663.6 | 2.24 | 0.115 |
| Arm | 58.0 | 1 | 58.0 | 0.2 | 0.660 |
| EQ5D VAS score at baseline | 1093.0 | 1 | 1093.0 | 3.69 | 0.060 |
| Residual | 17158.1 | 58 | 295.8 |  |  |
| Total | 18485.2 | 60 | 308.1 |  |  |

| ANCOVA at 6 month follow up |  |  |  |  |  |
| --- | --- | --- | --- | --- | --- |
| **Source of variation** | **SUM OF SQUARE** | **DF** | **MEAN SUM OF SQUARE** | **F-statistics** | **p-value** |
|  |  |  |  |  |  |
| Model | 1563.1 | 2 | 781.6 | 3.28 | 0.045 |
| Arm | 164.2 | 1 | 164.2 | 0.69 | 0.410 |
| EQ5D VAS score at baseline* | 1141.9 | 1 | 1141.9 | 4.79 | 0.033 |
| Residual | 13354.6 | 56 | 238.5 |  |  |
| Total | 14917.8 | 58 | 257.2 |  |  |

Repeated measures ANCOVA:

| Source of Variation | Coefficient | Standard Error | z-statistic | p-value | 95% CI | |
| --- | --- | --- | --- | --- | --- | --- |
| Between Individuals |  |  |  |  | **LL** | **UL** |
| Time | 0.00 | 0.00 |  |  | 0 | . |
| UID | 86.9 | 34.7 |  |  | 39.7 | 190.2 |
| Residual | 167.3 | 30.8 |  |  | 116.6 | 240.0 |
| Within Individuals |  |  |  |  |  |  |
| Time | 1.9 | 2.4 | 0.8 | 0.4 | -2.8 | 6.5 |
| Arm | -2.9 | 3.4 | -0.8 | 0.4 | -9.6 | 3.9 |
| EQ5D VAS Score at baseline | 0.3 | 0.1 | 2.5 | 0.0 | 0.1 | 0.5 |
| Intercept | 56.5 | 11.2 | 5.1 | 0.0 | 34.6 | 78.4 |
